# Harvesting of aerial humidity with natural hygroscopic salt excretions

**DOI:** 10.1073/pnas.2313134120

**Published:** 2023-10-30

**Authors:** Marieh B. Al-Handawi, Patrick Commins, Robert E. Dinnebier, Mahmoud Abdellatief, Liang Li, Panče Naumov

**Affiliations:** ^a^Smart Materials Lab, New York University Abu Dhabi, Abu Dhabi, United Arab Emirates; ^b^Max Planck Institute for Solid State Research, Stuttgart 70569, Germany; ^c^SESAME Synchrotron, As-Salt 19252, Jordan; ^d^Department of Sciences and Engineering, Sorbonne University Abu Dhabi, Abu Dhabi, United Arab Emirates; ^e^Center for Smart Engineering Materials, New York University Abu Dhabi, Abu Dhabi, United Arab Emirates; ^f^Research Center for Environment and Materials, Macedonian Academy of Sciences and Arts, MK-1000 Skopje, Macedonia; ^g^Department of Chemistry, Molecular Design Institute, New York University, New York, NY 10003

**Keywords:** biomineralization, extremopohiles, water collection, crystallization, deliquescence

## Abstract

Plants and animals that thrive in arid regions utilize the diurnal changes in environmental temperature and humidity to optimize their water budget by combining water-harvesting mechanisms and morphophysiological traits. The Athel tamarisk (*Tamarix aphylla*) is a halophytic desert shrub that survives in arid, hypersaline conditions by excreting concentrated solutions of ions as droplets on its surface that crystallize into salt crystals and fall off the branches. Here, we describe the crystallization on the surface of the plant and explore the effects of external conditions such as diurnal changes in humidity and temperature. The salt mixtures contain at least ten common minerals, with NaCl and CaSO_4_·2H_2_O being the major products, SiO_2_ and CaCO_3_ main sand contaminants, and Li_2_SO_4_, CaSO_4_, KCl, K_2_Ca(SO_4_)_2_·H_2_O, CaMg(CO_3_)_2_ and AlNaSi_3_O_8_ present in smaller amounts. In natural conditions, the hanging or sitting droplets remain firmly attached to the surface, with an average adhesion force of 275 ± 3.5 µN measured for pure water. Rather than using morphological features of the surface, the droplets adhere by chemical interactions, predominantly by hydrogen bonding. Increasing ion concentration slightly increases the contact angle on the hydrophobic cuticle, thereby lowering surface wettability. Small amounts of lithium sulfate and possibly other hygroscopic salts result in strong hygroscopicity and propensity for deliquescence of the salt mixture overnight. Within a broader context, this natural mechanism for humidity harvesting that uses environmentally benign salts as moisture adsorbents could provide a bioinspired approach that complements the currently available water collection or cloud-seeding technologies.

## Significance

Some of the most innovative water collection technologies are inspired by natural organisms that have optimized their physiology to thrive in arid conditions such as deserts. In this work, we explore the physicochemical aspects of salt release and water collection mechanisms by a desert shrub that has adapted to thrive in hypersaline sands. This work not only reveals the dual nature of the mechanisms underlying a plant’s ability to survive in highly saline regions but could also become an inspiration to devise methods for enhancement of the water-harvesting capacity of artificial materials by combining different principles of water collection and salt release.

The scarcity of fresh water has caused more than 663 million of the world’s population to currently live in water-stressed conditions, the majority being located in the developing regions of sub-Saharan Africa and Southern Asia (1). It is estimated that by 2025, about 1.8 billion people will experience severe water scarcity and hunger due to the loss of fertile farming lands caused by droughts (2). The ever-increasing water demand has stimulated research into alternative water-harvesting technologies to supplement the existing conventional resources in specific water-stressed locations that is poised to alleviate the foreseeable socioeconomic impacts of the inevitably increasing water scarcity. Fog and dew are natural, ever-present, abundant, and commonly available resources of water that hold an underexplored potential as an alternative water supply. In nature, this unconventional water source is utilized by a number of desert plants and animals, and the underlying principles of water collection provide an inspiration for emerging technologies that could potentially maximize the efficiency of the currently trialed artificial systems for the collection of aerial humidity. Various surface adaptations of xerophytic plants that thrive in arid environments are known to be capable of capturing fog and dew from the environment by both physical and chemical means. For instance, *Drimys brasiliensis* (3), some species of the *Crassula* genus (4), *Zygophyllum xanthoxylum* (5), and *Haloxylon ammodendron* (5) are all known to collect water by using porous and hydrophilic structures. Other plants, such as *Stipagrostis sabulicola* (6), *Syntrichia caninervis,* (7) and some *Setaria* grasses (8) have developed complex surface microstructures to condense and collect water in the vicinity of their roots. An additional level of sophistication to these water-collection mechanisms is provided by the recretohalophytes, a class of plants that thrive on highly saline soils and condense water using hygroscopic salts (9–11). The green foliage of these plants is decorated by salt glands that accumulate and excrete solutions rich in inorganic ions that have been absorbed through their roots (12). The solutions crystallize by water evaporation, and the resulting salt crystals are mechanically detached and released by the wind.

**Fig. 1.**
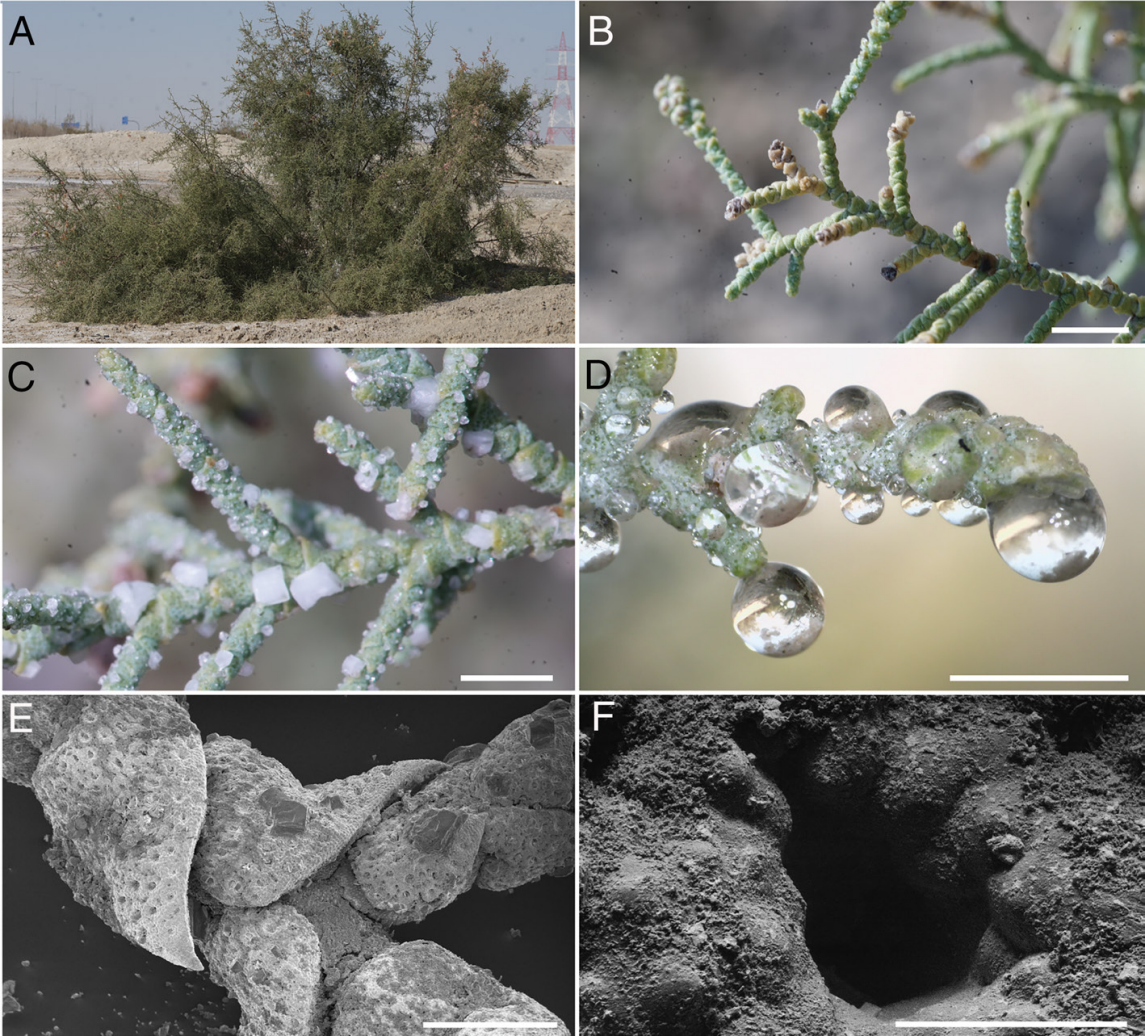
Appearance of the shrub, branches, and surfaces of *T. aphylla*. (*A*) A photograph of *T. aphylla* located across the coastal sabkha of the United Arab Emirates. (*B*) A close-up view of the branches. (*C*) A branch recorded in the late morning (11 a.m.) was encrusted with salt crystals. (*D*) A branch recorded in the early morning (8 a.m.) showed condensed water droplets. (*E* and *F*) Scanning electron microscopic images of the surfaces of leaves with salt crystals (*E*) and a zoomed image of a single salt gland (*F*). The length of the scale bars in panels *B*, *C*, *D*, *E*, and *F* are 5 mm, 3 mm, 3 mm, 1 mm, and 50 µm, respectively.

Some tamarisk (*Tamarix*) species, especially those distributed in the arid and semiarid regions in Europe, Africa, and Asia have been described as recretohalophytes (9, 10, 13). The genus comprises about 50 to 65 species (13, 14), with the Athel tamarisk (*T. aphylla*, Fig. 1*A*) being a typical species of this shrub native to the Middle East (14). The early studies have suggested that the halophytes excrete Na^+^ and Cl^−^ ions exclusively (15); however, subsequent detailed studies on various *Tamarix* species, including *T. aphylla*, revealed that the ion composition depends on the soil composition and the root environment (9, 10, 12, 16–20). The addition of monovalent and divalent ions to the soil results in the excretion of Na^+^, Rb^+^, Li^+^, K^+^, Mg^2+^, Ca^2+^, Si^4+^, Cl^−^, SO_4_^2−^, and CO_3_^2−^ by several *Tamarix* species (16–19). Another species, *T. smyrnensis*, has been demonstrated to be capable of releasing heavy metal ions such as Cd^2+^ and Pb^2+^ from contaminated soils (20–22). The excretion appears to be nonselective to the ion and occurs by ion exclusion from the salt glands (9, 12). Motivated by the capability of these plants to release inorganic salts and the potential significance that this desalination could have from a materials science perspective, here we delve into the details of the physicochemical crystallization and dissolution processes that occur on the surface of *T. aphylla*. Specifically, we reveal the effect of external conditions such as temperature and humidity on the crystallization and composition of the salts and the effect of the surface properties on the process. We also report an essential role of some of the inorganic crystals, which were found to be hygroscopic and deliquesce to adsorb and retain water on the surface. Altogether, our results unveil a complex interplay of mechanisms for salt release and humidity harvesting with an optimized combination of physiological, physicochemical, and morphological traits.

## Results and Discussion

Fig. 1 *A*–*F* shows optical and scanning electron microscopy (SEM) images of *T. aphylla* in the coastal sabkha (a term used for the coastal mudflat or sandflat that has been naturally enriched with salts under semiarid or arid climate conditions) of the United Arab Emirates. The succulent leaves are reduced to small scales that are 1 to 2 mm in height and are tightly wrapped around the stem (Fig. 1*B*). The leaves have a rough and uneven surface, and are typically encrusted with white particles in addition to sand. As shown in the zoomed-in images in Fig. 1 *E* and *F*, a dense distribution of salt glands is found along the shoot that appears as surface perforations. Close inspection of the leaves shows that they are decorated with white salt crystals having well-defined morphology (Fig. 1*C* and *SI Appendix*, Fig. S1). Our preliminary observations of the plant in its natural habitat indicated that during damp and foggy nights, the crystals deliquesce, resulting in the formation of small droplets (Fig. 1*D*). We monitored the diurnal cycle of salt excretion of *T. aphylla* (March 2019) by recording a time-lapse video of branches of the plant in the desert over 18 h. The ions excreted from the salt glands were found to undergo cycles of crystallization and dissolution, and the latter appeared to be facilitated by the deliquescence of the crystals.

**Fig. 2.**
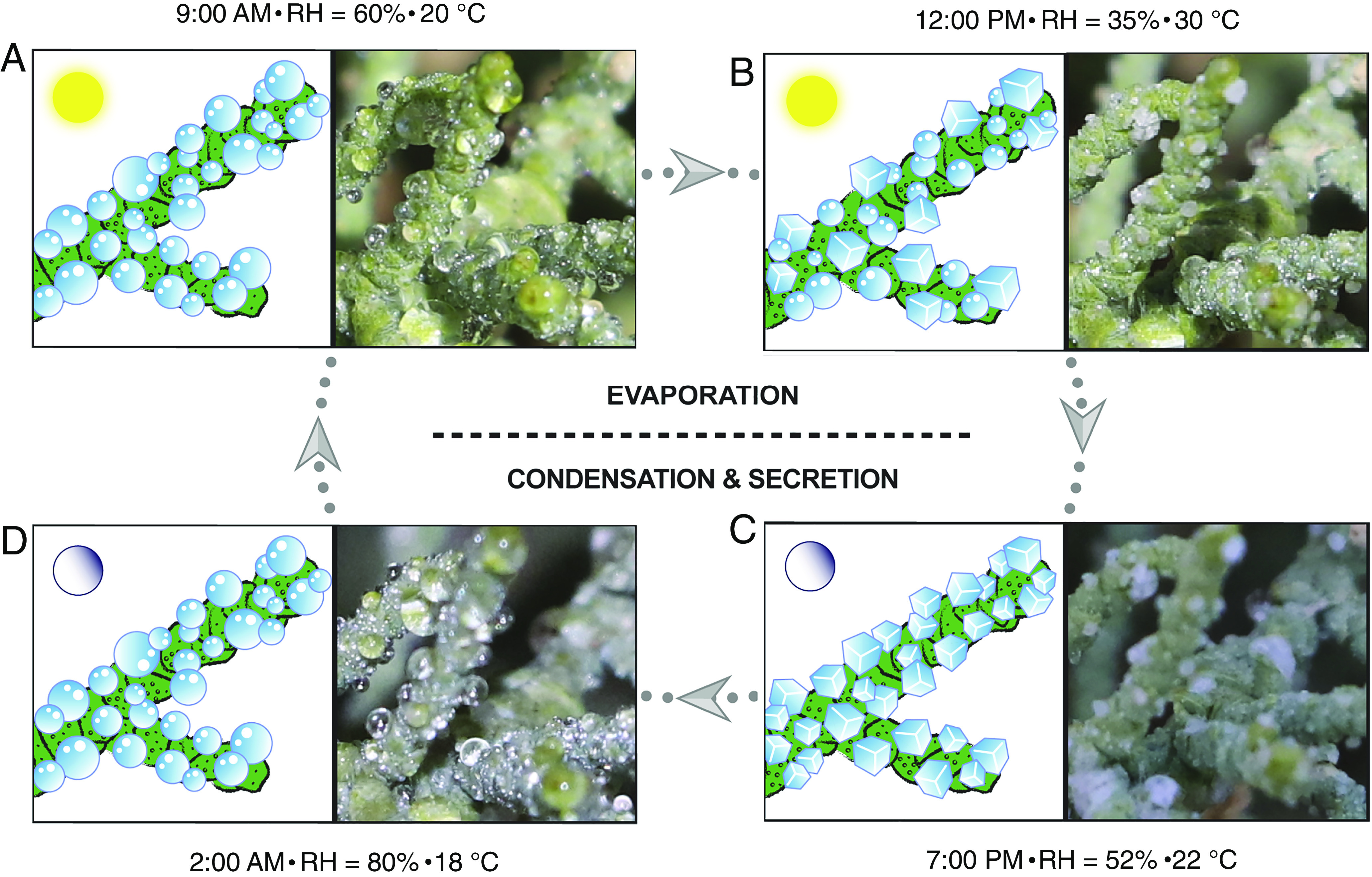
A summary of the water collection and salt crystallization over the diurnal cycle of *T. aphylla*. (*A*) The plant condenses water on its surface in the early morning when the humidity is high, and the temperature is low (9 a.m., 60% RH, 20 °C). (*B*) The water starts to evaporate, and the salts crystallize on the surface of the branch around midday, when the humidity is low and the temperature is high (12 p.m. noon, 35% RH, 30 °C). (*C*) During daytime, the water evaporates, resulting in the formation of salt crystals (7 p.m., 52% RH, 22 °C). (*D*) Overnight, when the humidity is high, the crystals adsorb water and dissolve (2 a.m., 80% RH, 18 °C).

As shown in Movie S1 and the snapshots extracted in Fig. 2, the leaf secretion occurs early in the morning and late at night. During the day, when the humidity is low, and the temperature is high, the water from the salt solution evaporates, resulting in the formation of crystals. In the morning, when the relative humidity (RH) and temperature are, for example, about 60% and 20 °C, droplets of excreted concentrated salt solution are clearly observed (Fig. 2*A*). As the humidity decreases and the temperature increases (35% RH, 30 °C), the water from the droplets evaporates, resulting in the formation of clumps of off-white crystals (Fig. 2 *B* and *C*). Over the night, the combination of very high humidity and low temperature (80% RH, ~18 °C) promotes the condensation of water onto the crystals and dissolution (Fig. 2*D*). The effect of the diurnal change in humidity and temperature monitored during a different time of the year (September 2019) confirmed that the cycle occurs via crystallization by evaporation and dissolution by deliquescence (Movie S2). It was also observed that occasionally sand particles carried by the wind settle on the branches, whereby the nucleation of the salt crystals in the hanging droplets is preferably initiated and occurs at the surface of the sand particles (*SI Appendix*, Fig. S1). In sitting droplets, on the other hand, both the sand particles and the surface of the cuticle wax act as nucleating sites. Nucleation of some salt crystals in sitting or hanging droplets was also observed near the edge of the droplets, probably due to the faster evaporation rate and higher concentration of solutes at the droplet interface compared to the bulk (23).

**Fig. 3.**
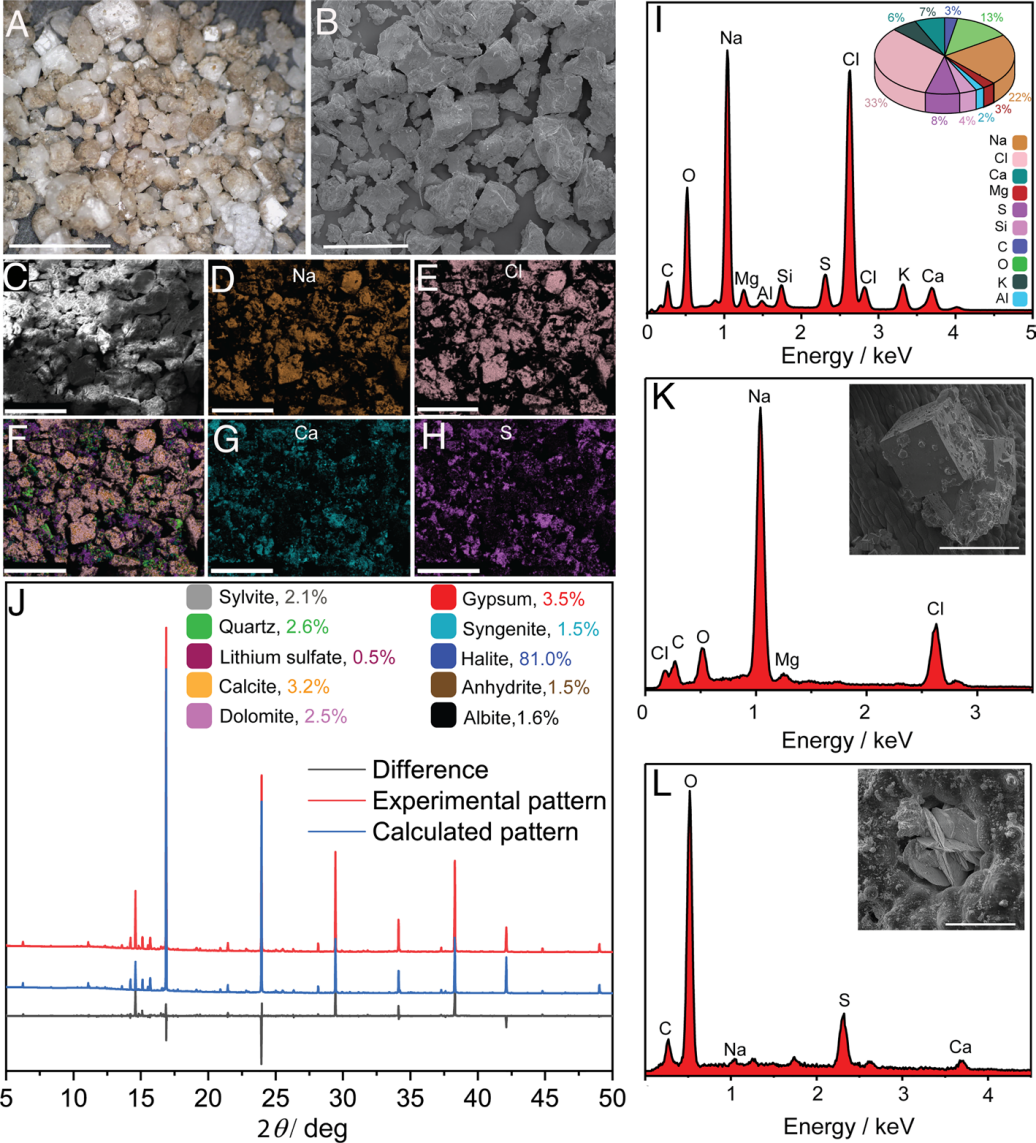
Characterization of the salts crystallized from the excretion of *T. aphylla*. (*A*) Optical image of the salt harvested from the branches. (*B*) Scanning electron micrograph (SEM) of the salt crystals, (*C*–*H* and *I*) SEM-EDX mapping and spectra of the plant salt. (*J*) Synchrotron PXRD pattern of the salt excreted by the plant and collected on May 2021 (sample ID: PS-4) (*K* and *L*) SEM images and EDX analysis of a cubic-shaped crystal (*K*) and a flower-shaped crystal (*L*). Scale bars for panels *A* and *B* are 5 mm and 500 μm, respectively, for *C*–*H* are 500 μm, and for the insets in panels *K* and *L* are 200 μm and 50 μm, respectively.

The salt crystals were mechanically isolated from the branch and analyzed (Fig. 3 *A* and *B*). The energy-dispersive X-ray spectra and elemental mapping established that the primary elements are Na (22%) and Cl (33%), with smaller amounts of K (6%), S (8%), Mg (3%), Ca (7%) and Si (4%) (Fig. 3 *C*–*I*). Overlap of the energy-dispersive X-ray analysis (EDX) maps of Na and Cl confirmed that the majority of the salt is composed of NaCl (Fig. 3*F*). These crystals are also the largest; they do not adhere well and are likely to easily fall off the branches under mechanical disturbance by the wind, which is an effective mechanism for the plant’s ability to remove most of the ions from its surface. The presence of other elements in relatively lower concentrations was also observed in regions that did not overlap with Na and Cl; these areas contained S, Mg, Ca, K, and Cl, suggesting the presence of alkali chlorides and sulfates of Mg, Ca, and K. X-ray photoelectron spectroscopy (XPS) measurements performed on three additional samples detected elemental composition similar to what was found by EDX (*SI Appendix*, Fig. S2 and Table S1) with additional traces of lithium in one of the samples (PS-5, *SI Appendix*, Fig. S2). To identify the exact salt composition, both synchrotron- and lab-based high-resolution powder X-ray diffraction (PXRD) analysis and Rietveld refinement were performed on a number of salt samples collected at various times of the year (*SI Appendix*, Figs. S3 and S4 and Tables S2 and S3). Fig. 3*J* shows as an example a PXRD pattern of a sample collected in May 2021 (sample ID: PS-4). The mixture was primarily composed (by mass) of halite (NaCl, 81.0%) with smaller amounts of gypsum (CaSO_4_·2H_2_O, 3.5%), sylvite (KCl, 2.1%), calcite (CaCO_3_, 3.2%), quartz (SiO_2_, 2.6%), anhydrite, (CaSO_4_, 1.5%), syngenite (K_2_Ca(SO_4_)_2_·H_2_O, 1.5%), dolomite (CaMg(CO_3_)_2_, 2.5%), albite (AlNaSi_3_O_8_, 1.6%), and lithium sulfate (Li_2_SO_4_, 0.5%).

With the excreted mixture of salts being a natural sample, we anticipated that its composition varies across the seasons, soil composition and plant physiology, among other factors, as it has been demonstrated previously (9, 10, 12, 15–20). High-resolution PXRD analysis of five salt samples that were collected at different seasons revealed that they contained at least eight common minerals, with NaCl, SiO_2_, CaCO_3_, and CaSO_4_·2H_2_O consistently being the major components by mass (*SI Appendix*, Table S2). Of these, the water-insoluble SiO_2_ and CaCO_3_ are attributed to contamination with sand particles that were insoluble and were expectedly found in higher concentrations on windy days (*SI Appendix*, Fig. S5). Crystals of NaCl and CaSO_4_·2H_2_O were the main products in the solid obtained by crystallization of the excreted liquid. SEM images of the crystals showed that the biggest white crystals have either cubic or stacked platy habits, and the EDX analysis confirmed that they were NaCl and CaSO_4_·2H_2_O, respectively (Fig. 3 *K* and *L*). Additional four minerals, namely Li_2_SO_4_, CaSO_4_, KCl, and K_2_Ca(SO_4_)_2_·H_2_O, were present in some of the samples in smaller amounts, and are hereafter referred to as minor components. Alongside high-resolution PXRD measurements, synchrotron PXRD was used to detect components in extremely low concentrations. A comparison between the laboratory and synchrotron PXRD analyses of three salt samples revealed the presence of at least three additional minerals, albite, dolomite, and aragonite, that were undetectable by laboratory-scale PXRD, and provided more accurate concentrations (*SI Appendix*, Table S3).

The ion composition of the water-soluble fraction on four different natural salt mixtures was also analyzed with mass spectrometry with inductively coupled plasma (ICP-MS) for the cations and with ion chromatography (IC) for the anions (samples PS-1, PS-3, PS-4, and PS-5; *SI Appendix*, Table S4). In all samples, the most abundant cation was Na^+^, with smaller amounts of K^+^, Mg^2+^, and Ca^2+^, and the predominant anions were Cl^─^ and SO_4_^2–^. The close molar concentrations of sodium and chloride as well as of calcium and sulfate are consistent with the PXRD results in that the mixture consists mostly of NaCl and CaSO_4_·2H_2_O. The excess chlorine, together with traces of other cations such as K^+^ and Mg^2+^, can be attributed to chlorides of these two counterions.

One of the central yet hitherto holistically unexplored aspects of the salt crystallization process of *T. aphylla* is the size, shape, and adhesion of the excreted ion-rich droplets onto the surface of its leaves. Some earlier observations from other xerophytes have indicated that the collected droplets fall from the branches to water the plant (6, 24). This, however, would be counterintuitive given that they are highly concentrated salt solutions. On the contrary, our careful observation and extended video recordings of excretion and crystallization with living *T. aphylla* plants in the desert showed that the salt solution's hanging or sitting droplets remain firmly attached to the surface. As illustrated by the sped-up recording in Movie S3 and the stills in Fig. 1*D*, the droplets do not fall off until the water completely evaporates and the crystals have formed and dried. Condensation was also observed at a high humidity level (~70% RH) in simulated conditions using an environmental SEM analysis on a branch of *T. aphylla* exposed to increasing humidity (*SI Appendix*, Fig. S6). As the droplets increased in volume, they remained attached to the surface due to the adhesive forces between the droplets and the waxy cuticle covering the leaves. These observations were unexpected in light of the anticipated hydrophobic nature of the cuticle covering the leaves (25).

The adhesion of the droplet to the surface can be facilitated by chemical forces or physical adhesion related to the hierarchical nanostructure of the surface (26). To deconvolute these two contributions, a model surface was prepared by spin-coating a solid surface (silicon wafer) with epicuticular wax extracted from the leaves of *T. aphylla*. As confirmed by using atomic force microscopy (AFM), the waxy layer formed a smooth surface with mean square roughness of <1 nm over an area of 0.5 × 0.5 µm, with the thickness of the layer varying across different regions (*SI Appendix*, Fig. S7). We note that the smoothness eliminates the possibility of the droplet being pinned by surface morphological features, such as, for example, those that the roughness would contribute, and therefore this model surface accounts only for the chemical interaction component. An explanation for the adhesion further required analysis of the extracted wax by using a combination of NMR spectroscopy, mass spectrometry (GC-MS), and infrared (IR) spectroscopy (*SI Appendix*, Figs. S8–S10 and Note 1). The analysis showed that the cuticle wax, a complex mixture of waxy compounds, contains both benzoate esters and long alkyl chains. The ester groups are capable of forming hydrogen bonds with water, and they would make it a likely candidate to explain the adhesive properties of the branch surface that holds the droplet attached.

**Fig. 4.**
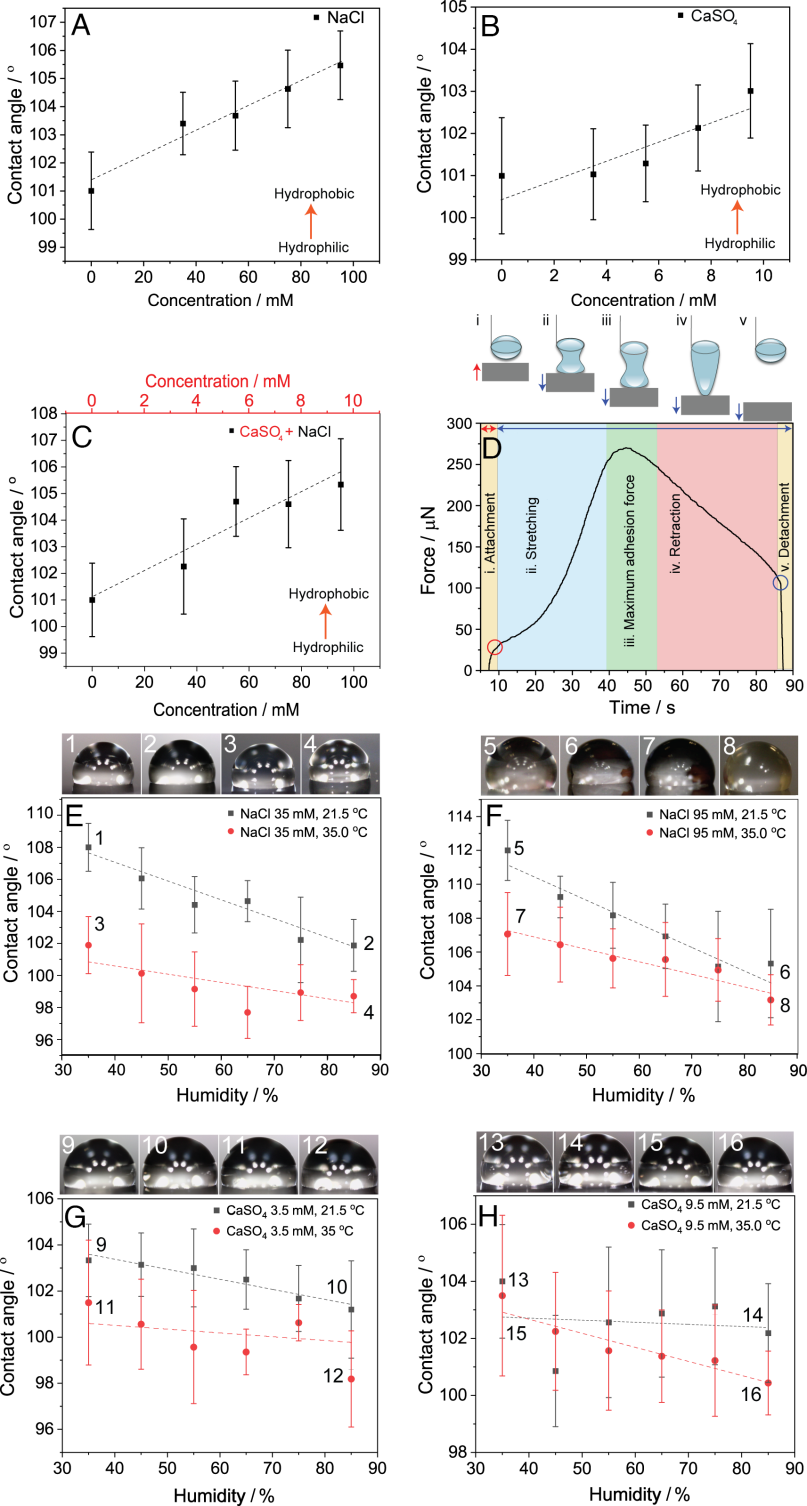
Dependence of the contact angle of salt solutions on salt concentration, humidity, and temperature. (*A*–*C*) The contact angle as measured for solutions of NaCl (*A*), CaSO_4_·2H_2_O (*B*), and NaCl mixed with CaSO_4_·2H_2_O (*C*) at different concentrations on a silicon wafer coated with epicuticular wax extracted from the leaves. (*D*) Adhesion force curves of a water droplet on the wax-coated silicon wafer. (i–v) schematic representation of the behavior of a water droplet on the surface of wax: (i) attachment of the droplet to the surface, (ii–iv) stretching of the droplet and the retraction of the stage, and (v) detachment of the droplet from the surface. (*E*–*H*) Contact angle measurements performed at different concentrations of NaCl [35 mM (*E*), 95 mM (*F*)] and CaSO_4_·2H_2_O [3.5 mM (*G*), 9.5 mM (*H*)], at two different temperatures, 21.5 °C (black) and 35 °C (red). The measurements were performed on a silicon wafer coated with the extracted epicuticular wax from the leaves. All measurements were performed under 35%, 45%, 55%, 65%, 75%, and 85% relative humidity (RH). Images of the droplets under various conditions are shown above each panel.

We analyzed the static contact angle on the model waxy surface to assess the surface wettability and, specifically, the effect of electrolytes. Since from the soluble components, it was concluded that the excreted salt mixture is typically composed mainly of NaCl and CaSO_4_·2H_2_O in approximately 10:1 molar ratio as an example for sample PS-3 (*SI Appendix*, Table S4), we measured the contact angles of droplets of aqueous solutions with different concentrations of each of the two salts as well as of their mixture. As shown in Fig. 4, the increasing concentration of NaCl results in a small increase in the contact angle from 100.8 ± 1.3° (pure water) to 105.5 ± 1.2° (95 mM NaCl). Within the same concentration range, the contact angle of CaSO_4_·2H_2_O follows a similar trend, with contact angles from 100.8° to 103.0°. Mixtures of NaCl and CaSO_4_·2H_2_O have contact angles between those of the pure salts, with the highest value being 105.6°. The small increase in the contact angle with increasing salt concentrations on hydrophobic surfaces has been reported previously (27, 28) and was explained by changes in the surface tension. Our results agree with these reports and show that higher electrolyte concentrations generally increase the contact angle on both hydrophilic and hydrophobic surfaces, although to a lesser extent in the latter case. Increasing electrolyte concentration is known to increase the surface tension of the droplet, which lowers its wettability, thereby increasing the contact angle (27–29).

To obtain a better insight into the adhesive properties of the epicuticular wax and to explain the retention of the droplets on the surface, we determined the adhesion force of a single water droplet (10 µL) on the wax-coated model surface as shown in Fig. 4*D*. The water droplet was placed on a platinum ring connected to a force balance. The force balance was raised till it made contact with the droplet. Once the droplet snapped onto the surface, it generated a force of 27 µN (point i in Fig. 4*D*). Then the force balance was lowered, causing the droplet to stretch and experience an adhesive force as it clung to the balance (point ii) and it reached a maximum adhesive force of 265 µN at 42 s (point iii). Beyond point iii, further stretching occurred, causing the contact area between the liquid-solid interface to decrease, lowering the force detected (point iv). At 85 s (point v) the pull-off force exceeded the adhesive force, and the droplet snapped off. The adhesion measurements were repeated three times on different areas of the sample, and the average adhesion force was 275 ± 3.5 µN (*SI Appendix*, Fig. S7*J*). As a benchmark, the adhesion force measurements were repeated three times in the same manner on Teflon, a known hydrophobic surface. The adhesion force was 155 ± 1 µN, which indicates that the wax of *T. aphylla* is approximately 1.8 times more adhesive to water than Teflon.

Since the relative humidity changes drastically during the diurnal cycle and can affect both the deliquescence point and the evaporation rate, we also tested the effect of relative aerial humidity on the contact angle. The measurements were performed with two concentrations of NaCl (95 mM, 35 mM) and CaSO_4_·2H_2_O (9.5 mM, 3.5 mM) at two temperatures that were selected to mimic the winter and summer conditions of the natural environment during the night (21.5 °C and 35 °C, respectively). At a constant temperature, the humidity varied from 35 to 85% in all experiments. The static contact angle for NaCl solution at a concentration of 35 mM decreased from 108.1° to 101.8° at 21.5 °C and from 101.8° to 98.7° at 35 °C, respectively (Fig. 4*E*). Similar trends of decreasing contact angles with increasing humidity were also found for solutions of NaCl and, and to a lesser extent, CaSO_4_, at different concentrations and temperatures (Fig. 4 *F*–*H*). This trend is due to the adsorption of water onto the waxy surface at high relative humidity, which results in higher surface energy and lower contact angle. These results conform to previous studies on the adhesion of water on hydrophilic and hydrophobic surfaces at varied humidity (30–32). The temperature increase from 21.5 °C to 35 °C results in decreased contact angle in most experiments due to the increased evaporation rate and decreased surface tension of the droplet (33, 34).

**Fig. 5.**
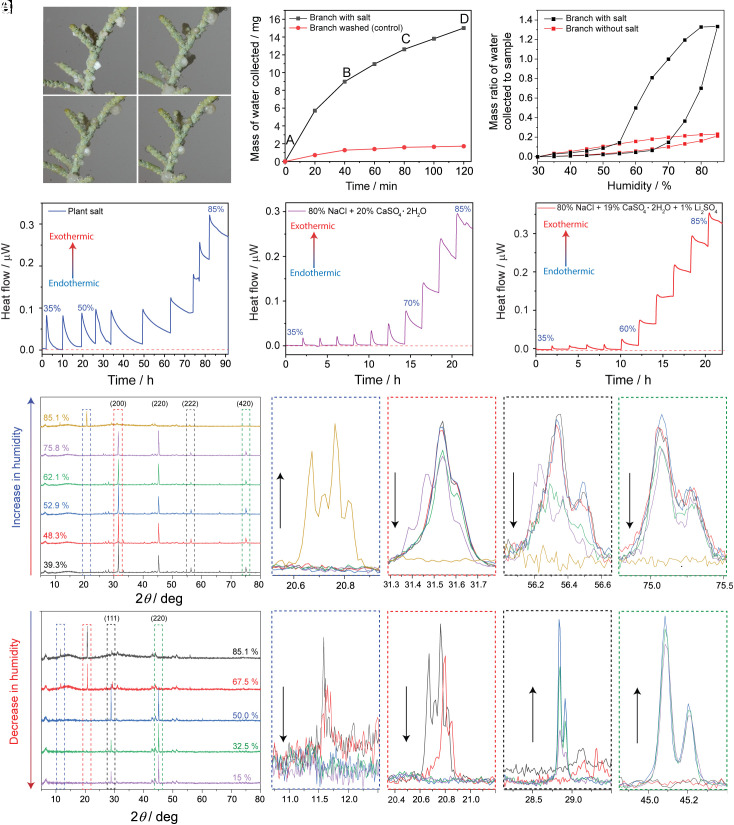
Water-collection and water-resorption ability of a freshly cleaved branch and the salt assessed by vapor sorption, calorimetry, and PXRD upon deliquescence. (*A*–*D*) Snapshots showing salt crystals attached to a branch deliquesce over time under high humidity (80%, 35 °C). (*E*) Mass of the water collected by the branch over time, together with a control branch where the salt crystals have been removed. (*F*) Ratio of the mass of water collected to mass of the sample as a function of thumidity. (*G*–*I*) Change in heat flow as a result in change of the relative humidity between 35 to 85% as a function of time of the plant salt (*G*), a model salt mixture composed of 80% NaCl and 20% CaSO_4_·2H_2_O (*H*), and a model mixture composed of 80% NaCl, 19% CaSO_4_·2H_2_O and 1% Li_2_SO_4_ (*I*). (*J*–*N*). Changes in the NaCl and CaSO_4_·2H_2_O diffraction peak intensities during the deliquescence and dissolution of the crystals induced by increase in humidity. (*O*–*S*) Dehydration and recrystallization of salts as humidity decreases from 85 to 15%. The sample in Fig. 5*E* was collected in September 2019. The analysis in Fig. 5*F* was performed on a different branch collected on May 2020. The analysis in Fig. 5*G* was performed on salt samples collected in January 2021 (PS-2). Fig. 5*J* shows the analysis of salt and branch collected in November 2020 (PS-1).

During our experiments, we observed that at increasing humidity, samples of the natural mixed salt visibly adsorbed moisture and began to deliquesce before they were dissolved in the adsorbed water (Fig. 5 *A*–*D*). To estimate the amount of water that the crystals can adsorb, the weight of a plant salt sample was measured while varying the humidity in an environmental chamber. A freshly cleaved branch (28 mg) was placed in the chamber which emulated the conditions in the desert during warm, damp nights in the summer. The relative humidity and temperature were set to 80% and 35 °C, respectively, and a video of the condensation process was recorded over 2 h, while also measuring the weight of the branch at 20-min intervals (Fig. 5*E*). It was found that after 2 h, the branch collected 15.2 mg of water. To verify that the condensation process was caused by the presence of the crystals and not by the surface of the leaves, the same branch was washed with deionized water to remove most of the salt crystals and dried for 1 h. The water-collecting ability was monitored over 2 h and only mild condensation was observed (1.6 mg of water collected), confirming that the hygroscopicity is due to adsorption of humidity by the crystals. The hygroscopicity was further quantified using dynamic vapor sorption analysis. The branch samples were equilibrated at 25 °C for an hour, followed by a 5% stepwise increase in humidity for 1 h. The isotherm depicted in Fig. 5*F* confirms that a clean branch (25 mg) can condense only 5 mg of water at 80% RH. A different branch of similar weight (30 mg) with crystals attached to its surface collected 40 mg of water at 80% RH, which is equivalent to 1.3 times the mass of the branch and an eightfold higher capacity for water collection compared to the clean branch. Moreover, the deliquescence of the solids on the branch with salt crystals starts at ~55% RH, which is much lower than the deliquescence points of both NaCl and CaSO_4_·2H_2_O.

The thermodynamics of adsorption of humidity and deliquescence point on natural salt mixtures was studied in detail by using precise microcalorimetric measurements. Equilibrated salt samples (25 °C, 35% RH) were exposed to increasing humidity in 5% steps every 2 to 6 h (depending on the time required for equilibration at each humidity level) between 35% and 85% to simulate the diurnal cycle in a desert environment. As shown in Fig. 5*G*, the native salt mixture does not adsorb water between 35% and 45% RH; instead, the adsorption of water starts around 50% RH, as indicated by the slightly positive increase in heat flow, and increases up to 85% RH. This process is reversible, and the decrease of RH to 35% results in partial evaporation. Since the combined chemical analysis of the native salt described above indicated that the major soluble components are NaCl (~80% w/w) and CaSO_4_·2H_2_O (~20% w/w), we prepared a simulant mixture composed of 80% NaCl and 20% of CaSO_4_·2H_2_O. Surprisingly, the simulant did not adsorb water in the 35 to 65% RH range and showed detectable adsorption only above 70% (Fig. 5*H*). This result is in accord with the deliquescence point of NaCl at ~70% RH and clearly does not originate from CaSO_4_·2H_2_O which does not deliquesces up to 99% RH (35). The lower deliquescence point of the native salt compared to the model mixture indicated that the natural mixture contains chemical components that decrease significantly the deliquescence point of the salt, while enhancing its hygroscopicity. The depression of the critical point for condensation (to ~50–55%) could be a mechanism by which *T. aphylla* maximizes condensation of water on its surface, which can then be reabsorbed through its leaves (*SI Appendix*, Fig. S11).

The depression of the deliquescence point suggested that the natural mixture contains one or more strongly hygroscopic minor components. A reference to the identified components of the natural salt and the deliquescent points of their various chemical forms available from the literature (*SI Appendix*, Table S2) indicates that Li_2_SO_4_, with deliquescence onset at ~60% RH determined in this work, is the only identified candidate that absorbs water at a lower humidity than NaCl. To validate this hypothesis, 1% of Li_2_SO_4_ was added to the salt simulant (NaCl and CaSO_4_·2H_2_O) and analyzed by microcalorimetry (Fig. 5*I*). Indeed, the presence of Li_2_SO_4_, even in such a minute amount, decreased the overall hygroscopicity of the simulant to 60% RH, close to that of the native salt. However, it should be noted that the presence of other, undetected hygroscopic salts with concentrations below the detection limit might also contribute to this effect, although based on our data (see below) Li_2_SO_4_ appears to be the most likely candidate. The stoichiometric excess of chlorine and magnesium in all samples and the overlap of the spatial distribution of chlorine with other elements such as magnesium and calcium in the atomic composition maps might indicate undetected amounts of potentially amorphous hygroscopic salts, such as MgCl_2_, CaCl_2_, LiCl, or K_2_CO_3_ (*SI Appendix*, Table S5), although the contribution to the overall hygroscopicity of such components is expected to be lower than that of Li_2_SO_4_. We note that our current study focuses on *T. aphylla* specimens that thrive in a specific region in the UAE (the coastal sabkha), and the results revealed that the salt mixtures are hygroscopic at low humidity (~55%) due to the presence of Li_2_SO_4_ in the soil. We anticipate the water adsorption ability of *T. aphylla* to vary across other locations since the hygroscopicity of the excreted salts depends on their composition, which is further determined by the composition of the soil, among other factors.

The changes in the crystallinity of the salt components during absorption of aerial humidity were monitored by using PXRD under controlled humidity (35 to 85%) at a constant temperature (25 °C). At low humidity (39% RH), the prominent diffraction peaks were from NaCl. Increase of the humidity to 52.9 to 62.1% resulted in the decrease of the diffraction peaks of NaCl [e.g., (200), (222) and (420); Fig. 5 *J*, *L*, *M*, and *N*], indicating partial deliquescence below the deliquescence point of NaCl (75% RH) (35) and are in line with the assumptions of the presence of another, more hygroscopic component. At 85% RH most of NaCl had dissolved, and only peaks from the minor components of low solubility, such as the (020) and (021) peaks of CaSO_4_·2H_2_O (99%) were observed (Fig. 5*K*). To monitor the crystallization, after the diffraction signature of NaCl at high humidity has disappeared, the humidity was decreased stepwise to 15% RH (Fig. 5*O*). The recrystallization of NaCl was detected at 50% RH, with the crystals preferably oriented on the (111) face, contrary to the previous experiment (increased humidity, Fig. 5*J*), where the preferred orientation was on the (200) face. It is worth noting that the intensity of the NaCl peaks should increase with a decrease in humidity as more NaCl recrystallizes from the solution. However, the intensity of the NaCl peaks was observed to decrease at 32.5% and 15% compared to 50% RH.

The significant depression of the humidity at which the excreted salts of *T. aphylla* start to adsorb water prompted us to consider the possibility of this mechanism as an alternative means for water collection from aerial moisture. Many plants, including halophytes that inhabit humid or arid regions, are known to have the ability of foliar uptake—absorption of water from the atmosphere through their foliage (3–5, 36–38). Studies performed on different halophytes such as *T. chinensis* (39), *Avicennia marina* (40), and *Nolana mollis* (24) revealed improvement in the plant’s water status by foliar uptake during precipitation events, fog condensation, and aerial water extraction by hygroscopic salts. However, these plants require high humidity levels (>75%) for foliar uptake. As described above, in our experiments, we observed that *T. aphylla* requires lower humidity levels of about 50 to 55% RH to initiate water adsorption and collection, which could play a role in the survivability of the plant. To confirm whether *T. aphylla* can uptake water through its aerial parts, 10 µL of 0.1% of aqueous solution of the dye lucifer yellow, an apoplastic tracer that tracks the movement of water within the leaves, was applied on the leaves of freshly extracted branches and left for ~7 h and subsequently removed by washing. A cross-section of the branch, against a nontreated branch as a control, shown in *SI Appendix*, Fig. S11, confirms that the dye has diffused through the cuticle and into the palisade and spongy cells of the treated leaf. The foliar uptake was observed with several leaves, although not all leaves exposed to the dye absorbed it. We attribute this result to the varying leaf water potential and status.

## Conclusions

In summary, we have demonstrated that the desert shrub *T. aphylla* excretes droplets that strongly adhere to its surface and crystallize into a mixture of salts that are capable of harvesting aerial water at significantly lower humidity levels (~50–55%) than the deliquescence point of the major constituents, NaCl and CaSO_4_·2H_2_O. A detailed study of the composition of the salts obtained after evaporation of the excreted liquid showed at least one minor strongly hygroscopic component (Li_2_SO_4_) that contributes to a significant expansion of the window of environmental conditions where the plant can harvest aerial water. We also provide evidence that the plant can absorb water by foliar uptake, which is enhanced by the hygroscopicity of the salt mixture and retention of the water on the surface. This enhanced water retention and harvesting mechanism differs from other reported xerophytes, which typically condense water from dew or fog through surface adaptations. Instead, *T. aphylla* relies almost entirely on chemical means to retain the secreted salt solutions in contact with its foliage, producing large salt crystals that can be removed by wind. Within a general context, this study not only reveals a unique natural complex mechanism for water utilization, but it also opens prospects for designing environmentally benign formulations based on a biogenic salt mixture that could be used for efficient harvesting of aerial water or cloud seeding at low humidity.

## Materials and Methods

### Chemicals and Reagents.

All commercially available chemicals salts and chemicals were purchased from Sigma-Aldrich and used without further purification. All solvents and reagents were used as received, without further purification.

### *T. aphylla* Sample Collection.

*T. aphylla* plants were located on the outskirts of Abu Dhabi near the region of Dhabeyya (recently renamed to Al Nouf) in the United Arab Emirates. The exact coordinates for the location for sample collection are 24°09′18.5″N, 54°16′06.6″E. The salt mixture was mechanically extracted from the branch and stored in vials containing silica beads to prevent water condensation. Branches of the plant were manually harvested and stored in plastic containers containing silica beads at room temperature.

### Time-Lapse Video Recording.

Snapshots of the process of salt formation and deliquescence on branches of the plant in its natural habitat were captured by using a Canon 80D camera with a time-lapse feature. The camera was set to capture an image every 2 min for a total time duration of 18 h. The images were collated to obtain a video recording.

### Powder X-ray Diffraction (PXRD) Analysis.

A Panalytical Empyrean diffractometer was used to record the PXRD powder diffraction patterns with Cu X-ray source (*λ* = 1.5418 Å) at power settings of 40 kV and 40 mA. A few milligrams of the *T. aphylla* salt were gently ground, and the powder was placed into a sample holder inside an environmental chamber. The temperature was fixed at 25 °C, and the humidity was increased in 5% increments. At each humidity level, the sample was left to equilibrate for 4 h before its diffraction pattern was recorded. The diffracted beam was measured using a Pixcel solid-state detector with a beta filter at a resolution of 0.0134°. For quantitative phase analysis, the measurements were carried out at room temperature on a Stoe Stadi P-diffractometer in Debye-Scherrer geometry with Mo *K*α_1_ radiation from primary Ge(111)-Johann-type monochromator and a Dectris 2 Mythen 1K silicon strip detector. The samples were either left untreated or carefully ground in an agate mortar before being loaded into 0.7-mm-diameter borosilicate capillaries (WJM-Glas/ Müller GmbH), which were spun during the measurement. The patterns were measured in a 2*θ* range from 0° to 73.0°. The total scan time was 10 to 12 h. Quantitative Rietveld refinement (41) of all crystalline phases was performed with the program TOPAS 7.0 (42). The peak profile was determined by a Pawley refinement (43), convoluted with the instrumental resolution function as determined from a line profile standard. The background was modeled using Chebyshev polynomials of higher order. Qualitative phase analysis was carried out by searching the ICDD-PDF4 database (44). The corresponding crystal structures of the identified phases were taken from the ICSD database (45). All crystalline phases down to about 1% could be identified.

For the high-resolution diffraction data, the salt powder was packed under nitrogen in 0.7-mm capillaries, and powder diffraction XRD data were collected using the MS beamline (46, 47) at the SESAME synchrotron at a wavelength of 0.82745 Å. The MS beamline is a wiggler-based beamline that uses two rhodium-coated mirrors and a double crystal Si (111) Kohzu monochromator, while the second crystal horizontal curvature is sagittal bendable. An ionization chamber in the experimental station was used to track the flux in time, which was used for the scale factor correction for long measurements. A two-circle vertical diffractometer was used, equipped with high-resolution encoders carrying a Pilatus 300 K detector of 172 µm pixel size at a distance of 740.4 mm from the sample. A Pilatus 300K area detector covering 6.4° at 740.4 mm distance from the sample was used to collect diffraction patterns from 3.0° to 80°, with an average exposure time of 5 min per frame. Borosilicate glass capillaries mounted on a standard goniometer head fixed on capillary spinner were used for the XRD measurements. The data were collected in transmission mode (Debye–Scherrer geometry) at room temperature. A NIST (640f) silicon standard was measured to calibrate the instrument while the lattice parameter of Si was used to determine the exact wavelength during the measurements. The detector was set to collect a diffraction image every 6°. The collected images were processed to extract the merged diffraction pattern using an ImageJ scripting mode.

### Scanning Electron Microscopy (SEM).

A fresh shoot of *T. aphylla* was affixed on an SEM stub using carbon tape and imaged under high vacuum mode by using an FEI Quanta 450 field-emission scanning electron microscope with a primary electron energy of 1 to 3 kV and a spot size of 1 to 3. EDX analysis was performed on crystals of different habits at an electron energy of 10 kV and with a scan duration of 150 s. The EDX mapping was performed with an electron energy of 15 kV and a scan duration of 2 h. For the environmental SEM experiments, a small section of the plant leaf was attached on a stub using carbon tape. The humidity was varied between 10% and 90% RH, and a video was recorded to capture the deliquescence of the salts at 15 kV.

### Adhesion Force Microscopy.

The interaction of deionized water with the *T. aphylla* wax and Teflon was analyzed on (Biolin) Attension Sigma force tensiometer 701. A hydrophilic platinum/iridium ring was used to hold a 10 μL droplet of deionized water. The force of the balance system was tared to zero, and the substrate (Teflon or plant wax) was placed on a motorized stage and raised up at a speed of 5 mm/s toward the water droplet. The surface was considered to be in contact when the force change was larger than 5 μN. The substrate surface continued to move up until the droplet snapped onto the surface, and after that the surface was retracted at a speed of 1.5 mm/s till the droplet snapped off.

### Nuclear Magnetic Resonance (NMR) Spectroscopy.

The NMR spectra of the *T. aphylla* wax were recorded at 25 °C on a 600 MHz (Bruker) Aeon 600 spectrometer for the ^1^H nuclei, ^13^C DEPT, ^1^H-^13^C HSQC, ^1^H-^13^C HMBC, and 125 MHz for the ^13^C NMR. The chemical shifts are reported in ppm relative to the signals corresponding to the residual nondeuterated solvent (CDCl_3_, 7.28 ppm).

### Contact Angle Measurements.

The *T. aphylla* cuticle (wax barrier) was extracted from the leaf using hexane (48). Approximately 500 mg of plant material (leaves and stem) was submerged in hexane for 2 min. The solvent was extracted, filtered, and evaporated to obtain the wax. In order to coat the silicon wafer with the wax, 1 mg of the wax was dissolved in 1 mL chloroform. A solution of 1 mg/mL of wax in chloroform was spin-coated at 1,500 rpm on a silicon wafer. The contact angle of deionized water on the surface was measured by dispensing a 2 μL droplet onto the surface of the substrate. High-contrast images of the droplets were captured using a Dinolite camera, and the contact angle values were manually extracted. The experiments under controlled temperature and humidity were performed in an environmental chamber (ETS) model 5503, where the temperature was set at 21 °C or 35 °C and the humidity level was varied between 35% and 85%. A DataPhysics OCA 15EC setup (sessile drop, manual fitting) was used to study the effect of the surface of the extracted wax on the contact angle of pure water and solutions with different concentrations of NaCl and CaSO_4_. The contact angle of the solutions was measured by dispensing 2 μL of droplet onto the surface of the wax. All experiments were repeated 6 to 10 times.

### Dynamic Vapor Sorption Analysis.

The hygroscopicity of *T. aphylla* branches was quantified using a Hiden isochema IGAsorp vapor sorption analyzer. A few milligrams of plant material was added to the holder (~30 mg), and the temperature was kept constant at 25 °C while the humidity level was varied between 35% and 85% in 5% steps every hour.

### Inductively Coupled Plasma Mass Spectrometry (ICP-MS) and Ion Chromatography.

A Perkin-Elmer OPTIMA DV7000 and Metrohm 930 Compact Flex were used to determine the concentration of ions in the salt. Plant salt was manually extracted from the branches, dissolved, and filtered through a membrane to remove the insoluble impurities such as sand. The purified plant salts were dissolved in 50 mL water and analyzed by using a calibration curve obtained with standard solution. The sample solution was diluted multiple times with water (7–15 dilutions) in order to obtain a concentration that would fit within the limits of the calibration curve.

### Gas Chromatography.

A PerkinElmer Clarus 610 gas chromatograph was utilized to analyze the composition of the extracted wax. One milligram of wax was dissolved in 1 mL chloroform, and the sample was injected into a fused silica column (SPB-624) at 300 °C for analysis.

### Fourier-Transform Infrared (FTIR) Spectroscopy.

The FTIR spectra were recorded in attenuated total reflection (ATR) absorbance mode by using an Agilent ATR 670 IR spectrometer. A few milligrams of the *T. aphylla* wax were deposited onto the ATR-FTIR cell. The scan was carried in the region 4,000–500 cm^−1^.

### Thermal Analysis.

The microcalorimetric analyses were performed on a TA TAM IV instrument with temperature and humidity control. Approximately 50 to 100 mg of plant salt or a model salt mixture was placed in the sample holder and left to equilibrate for 1 h at 25 °C and 35% RH. After the equilibration, the temperature was kept constant, while the humidity was increased stepwise by 5% after 2 to 6 h.

### Fluorescence Microscopy.

The confocal fluorescence imaging was performed on a Leica TCS SP8 microscope by using a PicoQuant PDL 880-B 40 MHz pulsed 405-nm diode laser as an excitation source. A section of the branch was extracted, and few droplets of 0.1% lucifer yellow were deposited on the leaves.

### Atomic Force Microscopy (AFM).

The AFM analysis was performed on a Bruker Dimension Icon AFM in tapping mode. The cantilever tip (ScanAsyst-Air) had a radius of 2 nm and a resonance frequency of 70 kHz. The acquired images were processed and analyzed using the Gwyddion software (49).

### X-ray Photoelectron Spectroscopy (XPS).

The XPS spectra were recorded using a Thermo Fisher Scientific Nexsa G2 XPS instrument. A few milligrams of the salt samples were mounted on conductive carbon tape inside the sample platter. Ten to fifteen scans were acquired for each sample with a spot size of 400 µm.

## Supplementary Material

Appendix 01 (PDF)

Movie S1. Time-lapse video of the *Tamarix aphylla* branches during the diurnal cycle showing the salt crystallization and dissolution cycle. The video was recorded with a plant in its natural habitat in March 2019.

Movie S2. Time-lapse video of the collection of humidity by the salt crystals of *Tamarix aphylla*. The video was recorded with a plant in its natural habitat in September 2019.

Movie S3. Salt crystallization from ionic excretions on the branches of the *Tamarix aphylla.*

## Data Availability

All study data are included in the article and/or supporting information.

## References

[1] UNICEF, Progress on Sanitation and Drinking Water: 2015 Update and MDG Assessment (World Health Organization, 2015).

[2] UNCCD, Desertification: The Invisible Frontline (United Nations Conventions to Combat Desertification, 2014).

[3] C. B. Eller, A. L. Lima, R. S. Oliveira, Foliar Uptake of fog water and transport belowground alleviates drought effects in the cloud forest tree species, *Drimys brasiliensis* (Winteraceae). New Phytol. **199**, 151–162 (2013). 23534879 10.1111/nph.12248

[4] C. E. Martin, D. J. von Willert, Leaf epidermal hydathodes and the ecophysiological consequences of foliar water uptake in species of *Crassula* from the Namib Desert in Southern Africa. Plant Biol. **2**, 229–242 (2000).

[5] X. Wang, H. Xiao, J. Ren, Y. Cheng, Q. Yang, An ultrasonic humidification fluorescent tracing method for detecting unsaturated atmospheric water absorption by the aerial parts of desert plants. J. Arid Land **8**, 272–283 (2016).

[6] A. Roth-Nebelsick , Leaf surface structures enable the endemic Namib desert grass *Stipagrostis sabulicola* to Irrigate Itself with fog Water. J. R. Soc. Interface **9**, 1965–1974 (2012). 22356817 10.1098/rsif.2011.0847 PMC3385753

[7] Z. Pan , The upside-down water collection system of *Syntrichia caninervis*. Nat. Plants **2**, 1–5 (2016). 10.1038/nplants.2016.76 27302768

[8] Y. Xue, T. Wang, W. Shi, L. Sun, Y. Zheng, Water collection abilities of green bristlegrass bristle. RSC Adv. **4**, 40837–40840 (2014).

[9] F. Yuan, B. Leng, B. Wang, Progress in studying salt secretion from the salt glands in recretohalophytes: How do plants secrete salt? Front. Plant Sci. **7**, 977 (2016). 27446195 10.3389/fpls.2016.00977 PMC4927796

[10] Y. Waisel, The glands of *Tamarix aphylla*: A system for salt recretion or for carbon concentration? Physiol. Plant. **83**, 506–510 (1991).

[11] N. Qvit-Raz, E. Jurkevitch, S. Belkin, Drop-size soda lakes: Transient microbial habitats on a salt-secreting desert tree. Genetics **178**, 1615–1622 (2008). 18245835 10.1534/genetics.107.082164 PMC2278082

[12] M. Dassanayake, J. C. Larkin, Making plants break a sweat: The structure, function, and evolution of plant salt glands. Front. Plant Sci. **8**, 406 (2017). 28400779 10.3389/fpls.2017.00406 PMC5368257

[13] H. Akhani, L. Mucina, The *Tamaricetea arceuthoidis*: A new class for the continental riparian thickets of the Middle East, Central Asia and the subarid regions of the Lower Volga Valley. Lazaroa **36**, 61–66 (2015).

[14] B. B. Baum, The Genus Tamarix (Israel Academy of Sciences and Humanities, 1978).

[15] P. F. Scholander, H. T. Hammel, E. Hemmingsen, W. Garey, Salt balance in mangroves 1. Plant Physiol. **37**, 722–729 (1962). 16655719 10.1104/pp.37.6.722 PMC406237

[16] R. Storey, W. W. Thomson, An X-ray microanalysis study of the salt glands and intracellular calcium crystals of *Tamarix*. Ann. Bot. **73**, 307–313 (1994).

[17] J. Hagemeyer, Y. Waisel, Excretion of ions (Cd^2+^, Li^+^, Na^+^ and Cl^−^) by *Tamarix aphylla*. Physiol. Plant. **73**, 541–546 (1988).

[18] H. Wilson, D. Mycock, I. M. Weiersbye, The salt glands of *Tamarix usneoides* E. Mey. ex Bunge (South African Salt Cedar). Int. J. Phytoremediation **19**, 587–595 (2017). 27739879 10.1080/15226514.2016.1244163

[19] W. W. Thomson, W. L. Berry, L. L. Liu, Localization and secretion of salt by the salt glands of *Tamarix aphylla*. Proc. Natl. Acad. Sci. U.S.A. **63**, 310–317 (1969). 16591764 10.1073/pnas.63.2.310 PMC223566

[20] E. Manousaki, J. Kadukova, N. Papadantonakis, N. Kalogerakis, Phytoextraction and phytoexcretion of Cd by the leaves of *Tamarix smyrnensis* growing on contaminated non-saline and saline soils. Environ. Res. **106**, 326–332 (2008). 17543928 10.1016/j.envres.2007.04.004

[21] J. Kadukova, E. Manousaki, N. Kalogerakis, Pb and Cd accumulation and phyto-excretion by salt cedar (*Tamarix smyrnensis* Bunge). Int. J. Phytoremediation **10**, 31–46 (2008). 18709930 10.1080/15226510701827051

[22] E. Manousaki, N. Kalogerakis, Halophytes—An emerging trend in phytoremediation. Int. J. Phytoremediation **13**, 959–969 (2011). 21972564 10.1080/15226514.2010.532241

[23] N. Shahidzadeh, M. F. L. Schut, J. Desarnaud, M. Prat, D. Bonn, Salt stains from evaporating droplets. Sci. Rep. **5**, 10335 (2015). 26012481 10.1038/srep10335 PMC4445064

[24] H. A. Mooney, S. L. Gulmon, J. Ehleringer, P. W. Rundel, Atmospheric water uptake by an Atacama Desert Shrub. Science **209**, 693–694 (1980). 17821192 10.1126/science.209.4457.693

[25] K. Koch, H.-J. Ensikat, The hydrophobic coatings of plant surfaces: Epicuticular wax crystals and their morphologies, crystallinity and molecular self-assembly. Micron **39**, 759–772 (2008). 18187332 10.1016/j.micron.2007.11.010

[26] H. Wang, H. Shi, Y. Li, Y. Wang, The effects of leaf roughness, surface free energy and work of adhesion on leaf water drop adhesion. PLoS One **9**, e107062 (2014). 25198355 10.1371/journal.pone.0107062 PMC4157819

[27] D. A. L. Leelamanie, J. Karube, Soil-water contact angle as affected by the aqueous electrolyte concentration. Soil Sci. Plant Nutr. **59**, 501–508 (2013).

[28] N. Sghaier, M. Prat, S. Ben Nasrallah, On the Influence of sodium chloride concentration on equilibrium contact angle. Chem. Eng. J. **122**, 47–53 (2006).

[29] E. Al-Zaidi, X. Fan, Effect of aqueous electrolyte concentration and valency on contact angle on flat glass surfaces and inside capillary glass tubes. Colloids Surf. A Physicochem. Eng. **543**, 1–8 (2018).

[30] J. L. Pérez-Díaz, M. A. Álvarez-Valenzuela, J. C. García-Prada, The effect of the partial pressure of water vapor on the surface tension of the liquid water-air interface. J. Colloid Interface Sci. **381**, 180–182 (2012). 22717083 10.1016/j.jcis.2012.05.034

[31] J. L. Perez-Diaz , “On the influence of relative humidity on the contact angle of a water droplet on a silicon wafer” in ASME 2013 International Mechanical Engineering Congress and Exposition (American Society of Mechanical Engineers, New York, NY, 2013).

[32] X. Xiao, Y. T. Cheng, B. W. Sheldon, J. Rankin, Condensed water on superhydrophobic carbon films. J. Mater. Res. **23**, 2174–2178 (2008).

[33] J. K. Park, J. Ryu, B. C. Koo, S. Lee, K. H. Kang, How the change of contact angle occurs for an evaporating droplet: Effect of impurity and attached water films. Soft Matter **8**, 11889–11896 (2012).

[34] J.-W. Song, L.-W. Fan, Temperature dependence of the contact angle of water: A review of research progress, theoretical understanding, and implications for boiling heat transfer. Adv. Colloid Interface Sci. **288**, 102339 (2021). 33385775 10.1016/j.cis.2020.102339

[35] T. D. Gonçalves, J. D. Rodrigues, M. M. Abreu, Evaluating the salt content of salt-contaminated samples on the basis of their hygroscopic behaviour: Part II: Experiments with nine common soluble salts. J. Cult. Herit. **7**, 193–200 (2006).

[36] K. Steppe , Direct uptake of canopy rainwater causes turgor-driven growth spurts in the mangrove *Avicennia marina*. Tree Physiol. **38**, 979–991 (2018). 29562244 10.1093/treephys/tpy024

[37] S. Li, H. Xiao, L. Zhao, M.-X. Zhou, F. Wang, Foliar water uptake of *Tamarix ramosissima* from an atmosphere of high humidity. Sci. World J. **2014**, e529308 (2014). 10.1155/2014/529308 PMC4058515 24982964

[38] Z. C. Berry, N. C. Emery, S. G. Gotsch, G. R. Goldsmith, Foliar water uptake: Processes, pathways, and integration into plant water budgets. Plant Cell Environ. **42**, 410–423 (2019). 30194766 10.1111/pce.13439

[39] T. Hussain , The presence of salts in the leaf exudate improves the photosynthetic performance of a recreto-halophyte, Tamarix chinensis. Environ. Exp. Bot. **199**, 104896 (2022).

[40] R. E. Coopman , Harvesting water from unsaturated atmospheres: Deliquescence of salt secreted onto leaf surfaces drives reverse sap flow in a dominant arid climate mangrove, Avicennia marina. New Phytol. **231**, 1401–1414 (2021). 33983649 10.1111/nph.17461

[41] H. M. Rietveld, A profile refinement method for nuclear and magnetic structures. J. Appl. Cryst. **2**, 65–71 (1969).

[42] A. A. Coelho, TOPAS and TOPAS-Academic: An optimization program integrating computer algebra and crystallographic objects written in C++. J. Appl. Cryst. **51**, 210–218 (2018).

[43] G. S. Pawley, Unit-cell refinement from powder diffraction scans. J. Appl. Cryst. **14**, 357–361 (1981).

[44] S. D. Gates-Rector, T. N. Blanton, The powder diffraction file: A quality materials characterization database. Powder Diffr. **34**, 352–360 (2019).

[45] G. Bergerhoff, I. D. Brown, Crystallographic Databases (International Union of Crystallography, Chester, 1987).

[46] M. Abdellatief , Operational status of the X-ray powder diffraction beamline at the SESAME synchrotron. J. Synchrotron Rad. **29**, 532–539 (2022). 10.1107/S1600577521012820 PMC8900843 35254318

[47] M. Abdellatief , The SESAME materials science beamline for XRD applications. Powder Diffr. **32**, S6–S12 (2017).

[48] M. Saber, M. A. Kashmiri, A. Mohy-ud-din, M. Ahmed, R. Khanum, Epicuticular wax of Tamarix aphylla L. J. Chem. Soc. Pak. **32**, 662–667 (2010).

[49] D. Nečas, P. Klapetek, Gwyddion: An open-source software for SPM data analysis. Cent. Eur. J. Phys. **10**, 181–188 (2012).

