# Supplementary material for: Harvesting of aerial humidity with natural hygroscopic salt excretions
Source: Proc Natl Acad Sci U S A. 2023 Oct 30;120(45):e2313134120. doi: 10.1073/pnas.2313134120 (PMC10636306; doi:10.1073/pnas.2313134120)
Supplement: Supplementary file 1 — Appendix 01 (PDF) [file pnas.2313134120.sapp.pdf]

PNAS

Supporting Information for

Harvesting of Aerial Humidity with Natural Hygroscopic Salt Excretions

Marieh B. Al-Handawi[1], Patrick Commins[1], Robert E. Dinnebier[2], Mahmoud Abdellatief[3], Liang Li[1,4*],and Panče Naumov[1,5,6,7*]

[1]Smart Materials Lab, New York University Abu Dhabi, POB 129188, Abu Dhabi, United Arab Emirates
[2]Max Planck Institute for Solid State Research, Heisenbergstrasse 1, 70569 Stuttgart, Germany
[3]SESAME Synchrotron, King Hussein Bin Talal St, Allan 19252, Jordan
[4]Department of Sciences and Engineering, Sorbonne University Abu Dhabi, POB 38044, United Arab Emirates
[5]Center for Smart Engineering Materials, New York University Abu Dhabi, POB 129188, Abu Dhabi, United Arab Emirates
[6]Research Center for Environment and Materials, Macedonian Academy of Sciences and Arts, Bul. Krste Misirkov 2, MK–1000 Skopje, Macedonia
[7]Molecular Design Institute, Department of Chemistry, New York University, 100 Washington Square East, New York, NY 10003, United States
*Authors for correspondence: Liang Li, Panče Naumov
Emails: liang.li@sorbonne.ae (L.L.), pance.naumov@nyu.edu (P.N.)

This PDF file includes:

Supporting Information Notes
Figures S1 to S11
Tables S1 to S5
Legends for Movies S1 to S3

Other supporting materials for this manuscript include the following:

Movies S1 to S3

Supporting Information Notes

Supporting Information Note 1. The chemical composition of the plant wax was analyzed by using 1D and 2D NMR (Fig. S8 and S9). The ^{1}H NMR of the sample showed several complex multiplets between 1.6 and 0.9 ppm, which is a characteristic of natural waxes[1]. The aromatic region of the spectra showed a doublet and two triplets at 8.1, 7.6 and 7.5 ppm respectively. They integrate in a 2-to-1-to-2 integration ratio, which indicates the presence of a monosubstituted aromatic ring. The spectrum noticeably lacks any strong signals at ~2.1 ppm, which is common for benzylic protons, and this suggests that a quaternary functional group lies at that position. The ^{13}C NMR confirms the presence of a monosubstituted aromatic ring with four signals at 132.8, 130.6, 129.5 and 128.3, and the quaternary carbons showed an expected lower intensity than the other three signals. There is also a weak signal at 166.7 ppm, which we have assigned to an ester functional group. Additionally, there are many peaks at <40 ppm attributed to the various carbons associated with the long alkyl chains of the waxy molecules. The ^{1}H-^{13}C HSQC and HMBC experiment further clarified the structure by confirming the compound as a waxy benzylic ester. The HMBC showed a strong cross peak between the ester carbon 5 and the aromatic carbon 3, which proved the ester was at the benzylic position. The HMBC cross peak of carbon 5 to carbon 6 also allowed us to identify which carbon was nearest to the benzylic ester oxygen. The ^{13}C DEPT 135 NMR showed an upwards peak for carbon 6, and it was assigned as a secondary carbon (Fig. S8). Ideally, we would have been able to use HSQC and HMBC to determine the number of alkyl chain carbons in the benzylic ester, however, the spectral overlap between the benzylic ester and other waxes in the alkyl part of the spectra was too intense and we were unable to determine the chain length. However, the information from the ^{1}H and ^{13}C NMR in conjunction with the high-resolution mass spectrometric data (Fig. S10) allowed us to identify the molecules as waxy benzylic esters.

Supporting Information Figures

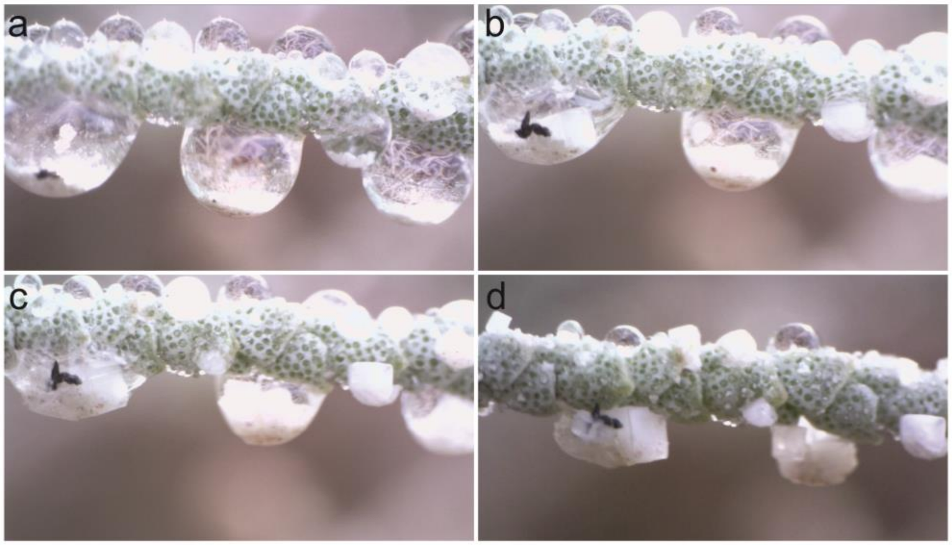

Fig. S1. Optical images of the crystallization of salts on the surface of a *T. aphylla* branch recorded at (a) 9 am, (b) 10 am, (c) 12 pm, and (d) 1 pm.

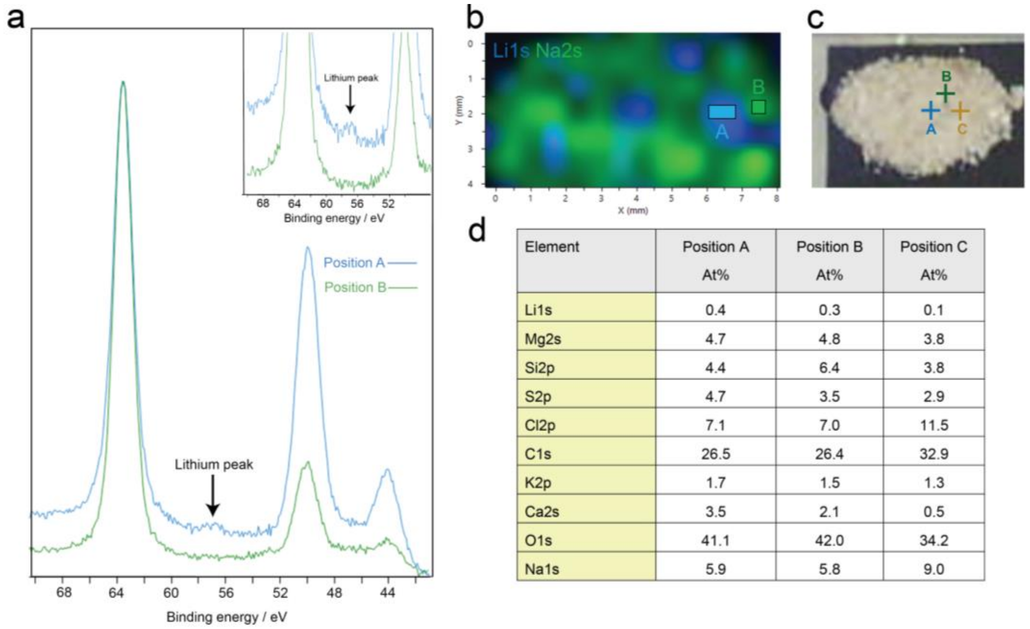

Element	Position A At%	Position B At%	Position C At%
Li1s	0.4	0.3	0.1
Mg2s	4.7	4.8	3.8
Si2p	4.4	6.4	3.8
S2p	4.7	3.5	2.9
Cl2p	7.1	7.0	11.5
C1s	26.5	26.4	32.9
K2p	1.7	1.5	1.3
Ca2s	3.5	2.1	0.5
O1s	41.1	42.0	34.2
Na1s	5.9	5.8	9.0

Fig. S2. XPS analysis on the *T. aphylla* salt (sample PS-5). (a) XPS spectra at two different regions of the salt sample, and (b) a peak intensity map. The areas marked A and B are the locations where the spectra shown in panel a have been collected. (c) Optical image of the sample used for XPS analysis. (d) Elemental analysis of three different locations in the sample marked in panel c.

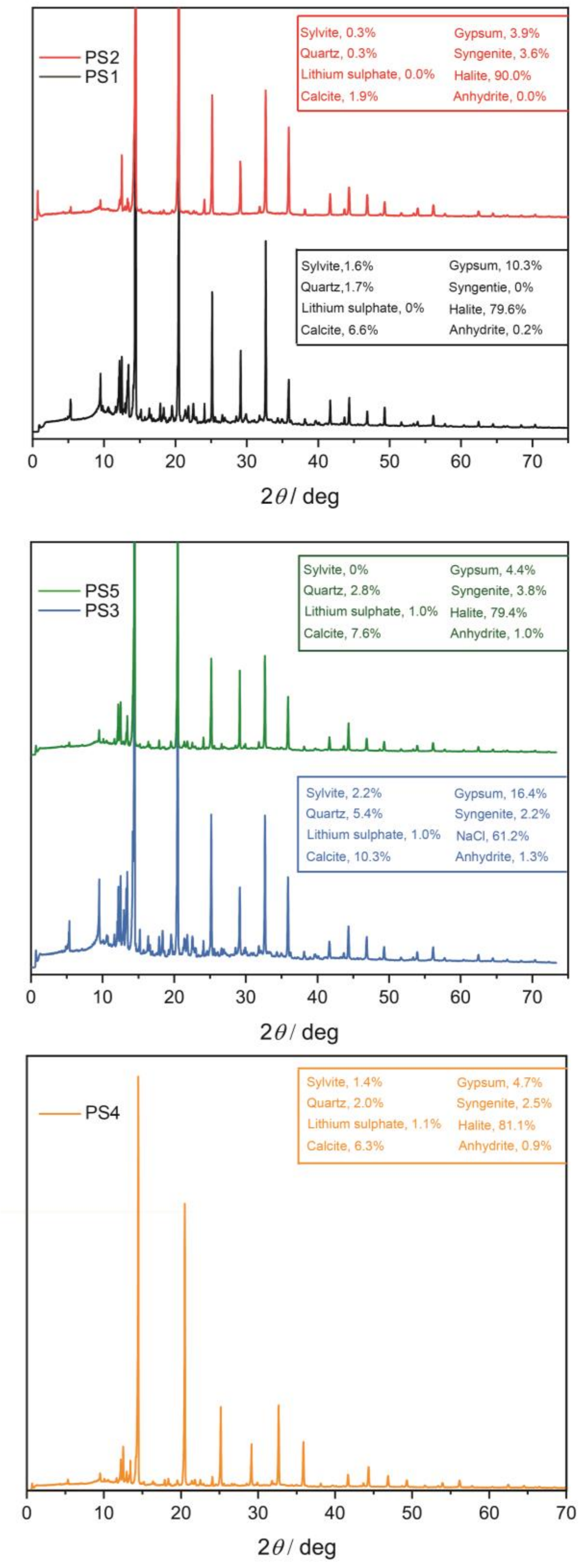

Fig. S3. High-resolution powder X-ray diffraction (PXRD) patterns and the respective compositional difference between five different natural samples of *T. aphylla* salt collected during different times of the year.

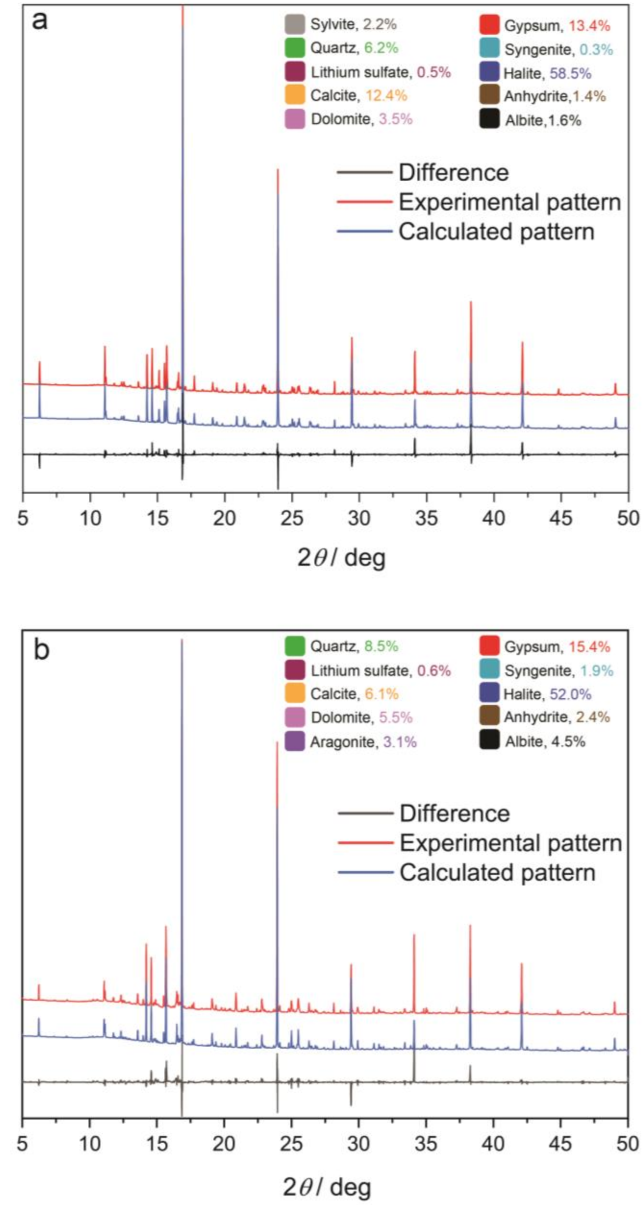

Fig. S4. Synchrotron powder X-ray diffraction (PXRD) patterns, Rietveld plots, and composition of two different natural samples of *T. aphylla* salt collected during different times of the year. Samples: (a) PS-3, (b) PS-5.

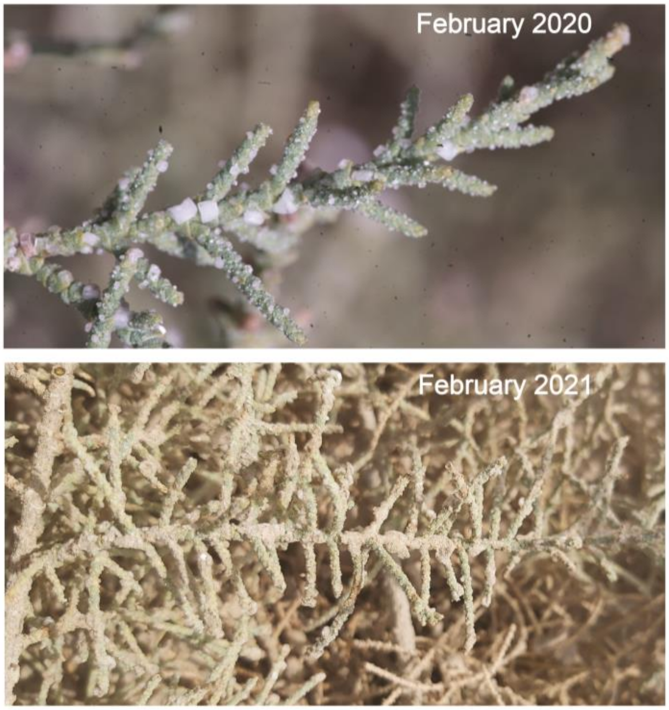

Fig. S5. Photographs of branches recorded in February 2020 and February 2021, the latter date coincided with a sandstorm occurrence. Following the sandstorm, a salt sample was extracted from the branch and subjected to analysis (PS-3), which revealed significantly higher concentrations of sand components ($CaCO_3$ and SiO_2).

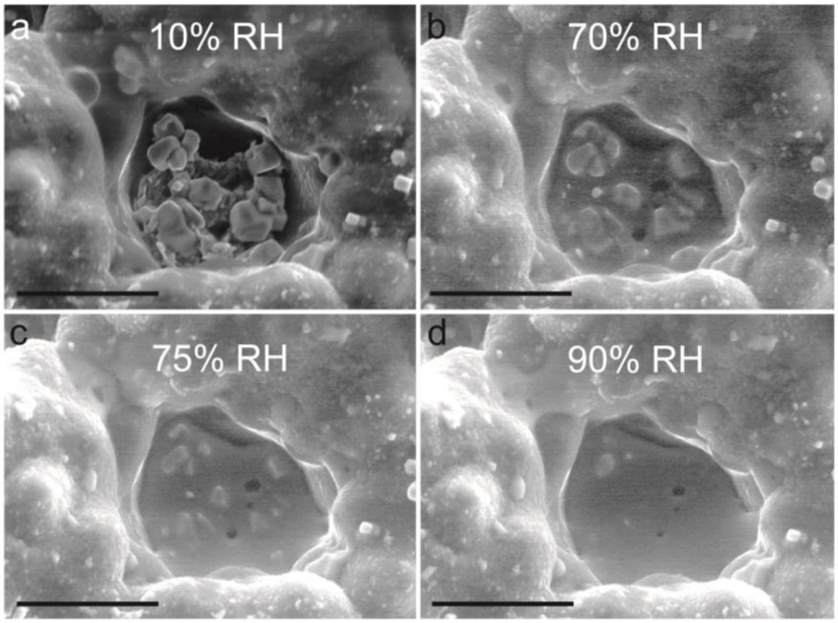

Fig. S6. Environmental scanning electron microscopy (ESEM) images of a single salt gland with salt crystallized in its cavity. The humidity was varied between (a) 10%, (b) 70%, (c) 75% and (d) 90% RH to monitor the deliquescence process. The scale bar in all images is 40 μm.

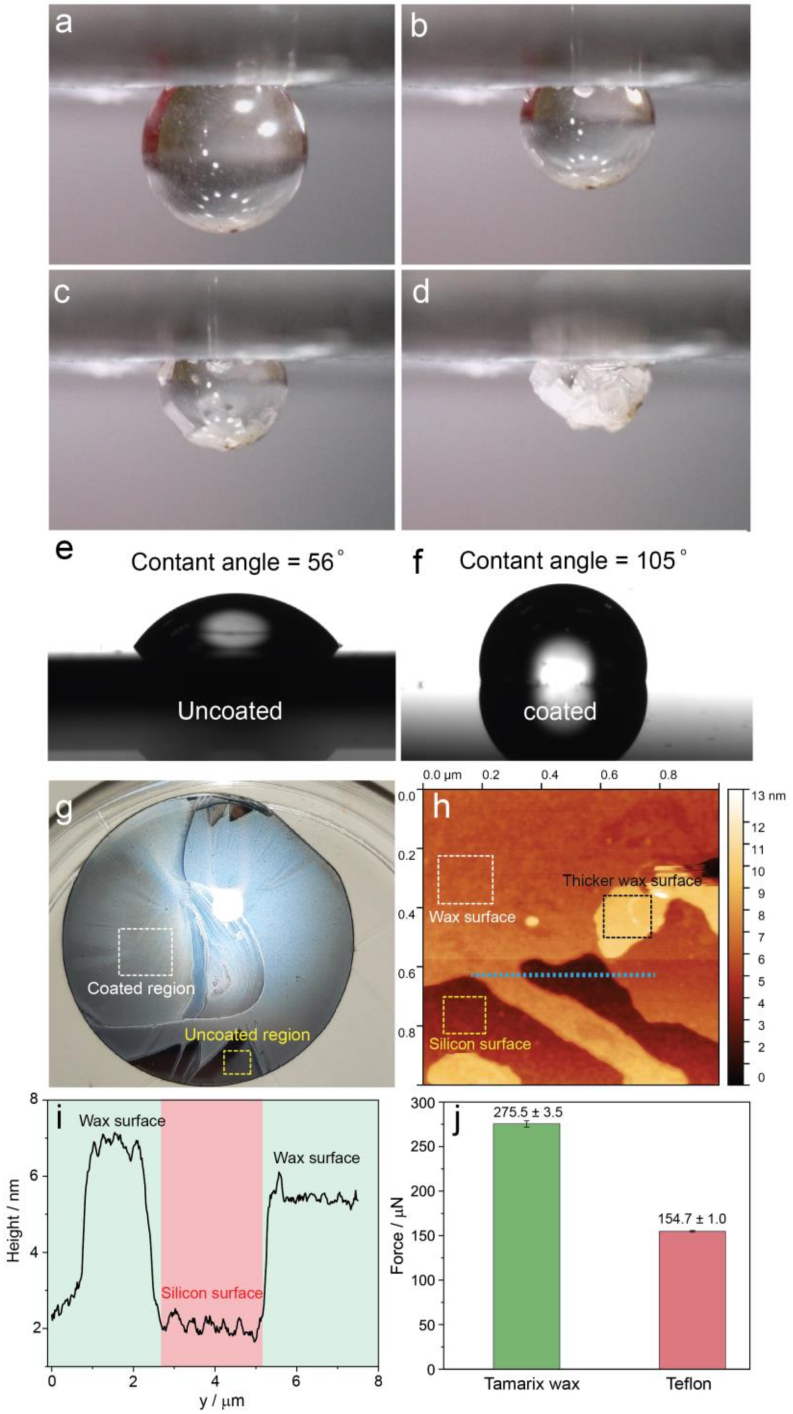

Fig. S7. Crystallization of the salts and relation to the surface properties. (a–d) Optical images of a hanging droplet of saline solution excreted from the plant on a silicon wafer coated with the plant wax. Contact angle measurements on (e) an uncoated silicon wafer and (f) coated silicon wafer. (g) Optical image of the plant wax deposited on a silicon wafer. (h) AFM topology image of *T. aphylla* wax deposited on a silicon wafer. (i) 2D height profile extracted from the AFM image shown in panel h, highlighted as a blue line. (j) Average adhesion force measurements with the standard deviations (n = 3) of a pure water droplet (10 μL) on the wax extracted from the surface of the plant and coated on a silicon wafer (green) and on a Teflon surface (red).

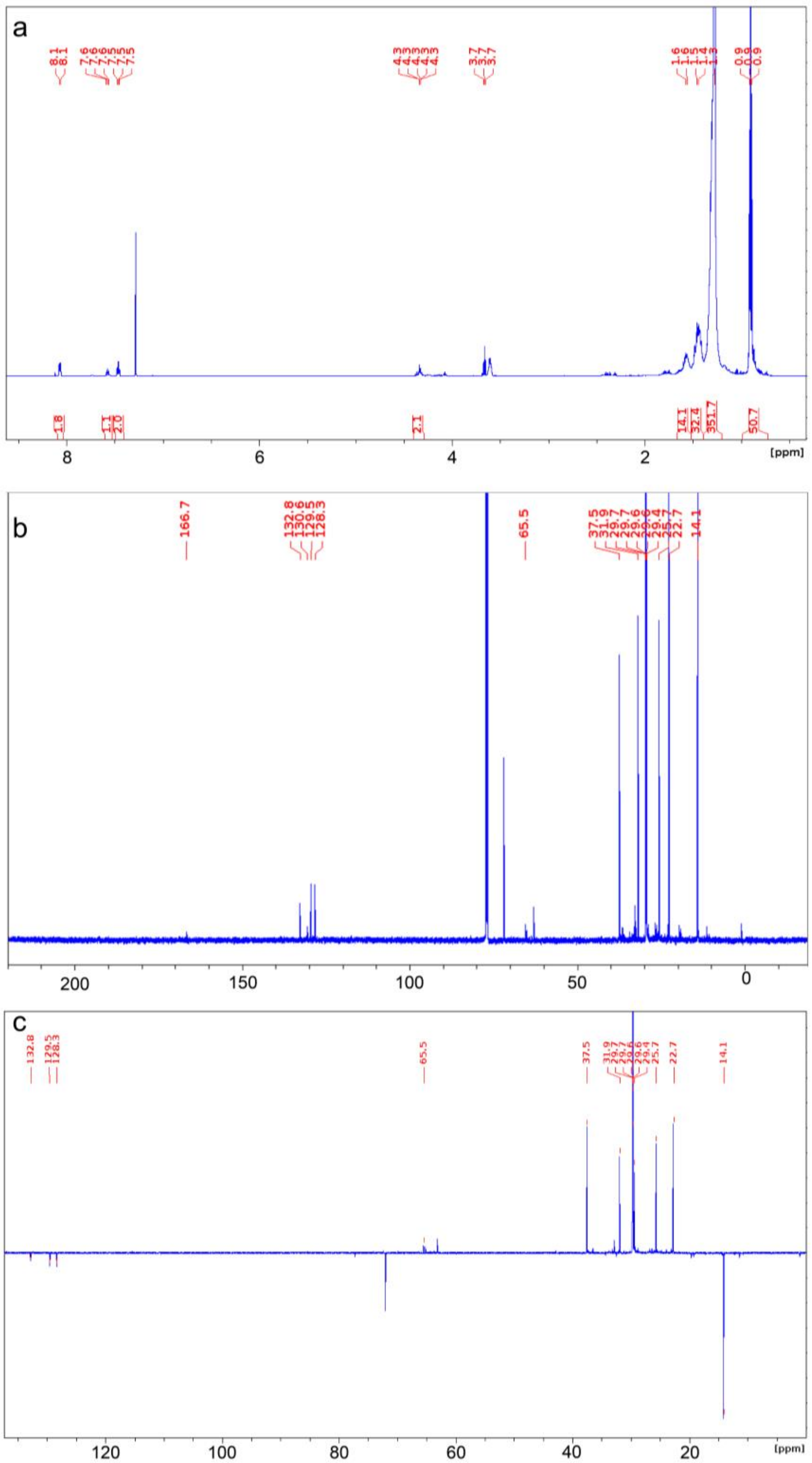

Fig. S8. 1D NMR analysis of the of plant wax in $CDCl_3$. (a) ^{1}H NMR spectrum (500 MHz), (b) ^{13}C NMR spectrum (125 MHz), and (c) ^{13}C DEPT 135 NMR spectrum (125 MHz).

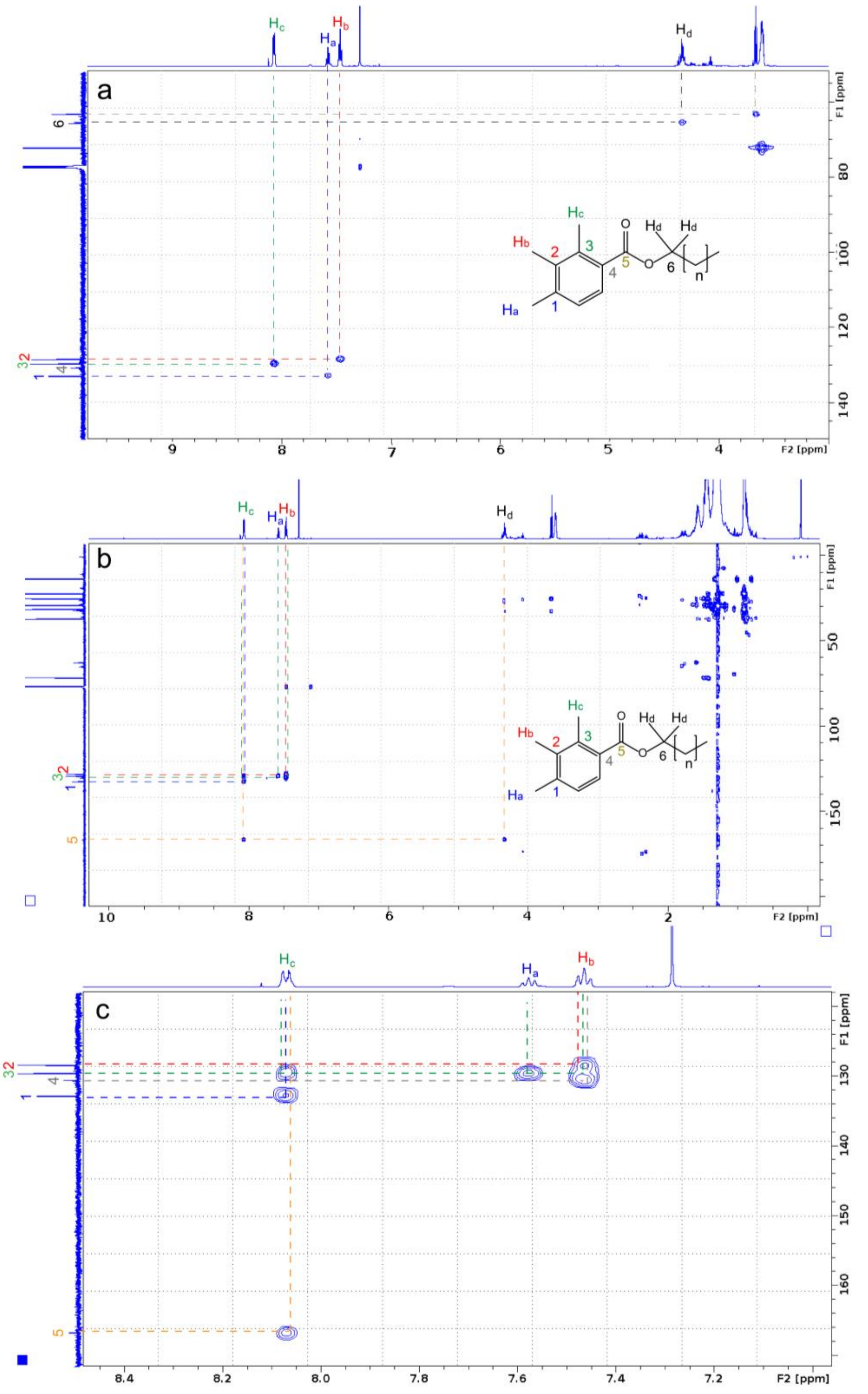

Fig. S9. 2D NMR analysis of the plant wax. (a) ^{1}H-^{13}C HSQC NMR of the plant wax in $CDCl_3$ (500 MHz), (b) ^{1}H-^{13}C HMBC NMR spectrum (500 MHz) of the plant wax in $CDCl_3$ (c) Magnified region of ^{1}H-^{13}C HMBC NMR spectrum (500 MHz).

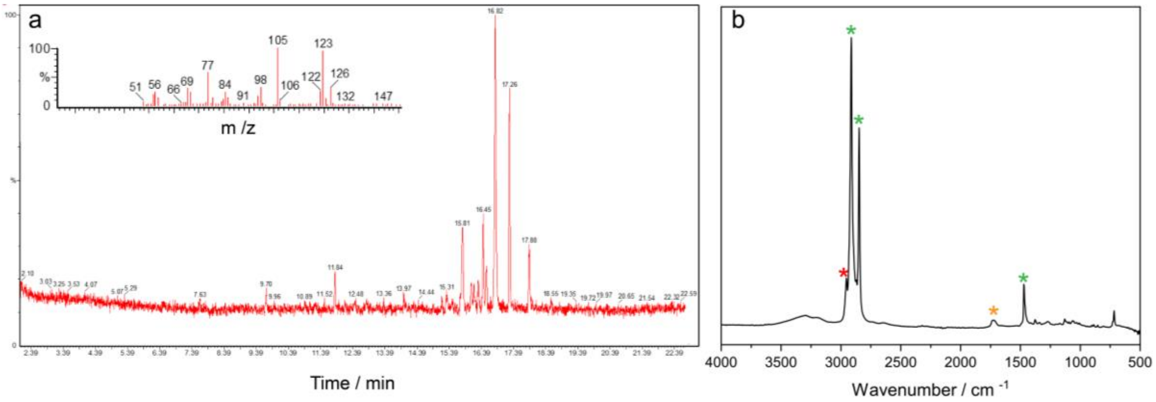

Fig. S10. Mass spectrometric analysis of the wax. (a) GC-MS spectrum of the leaf wax. The inset represents the mass spectrum with significant ions of m/z 123, 105 and 77, characteristic for benzoic esters[2]. (b) ATR-IR spectrum of the extracted leaf wax. The peak at 2956 cm^{-1} (red asterisk) represents the aromatic C—H stretch, while the peaks at 2849 and 2918 cm^{-1} (green asterisks) are attributed to the aliphatic alkyl C—H stretch. The peak at 1735 cm^{-1} (yellow asterisk) is from a C=O stretch, and the peak at 1472 cm^{-1} (green asterisk) could be assigned to the C—H scissoring mode.

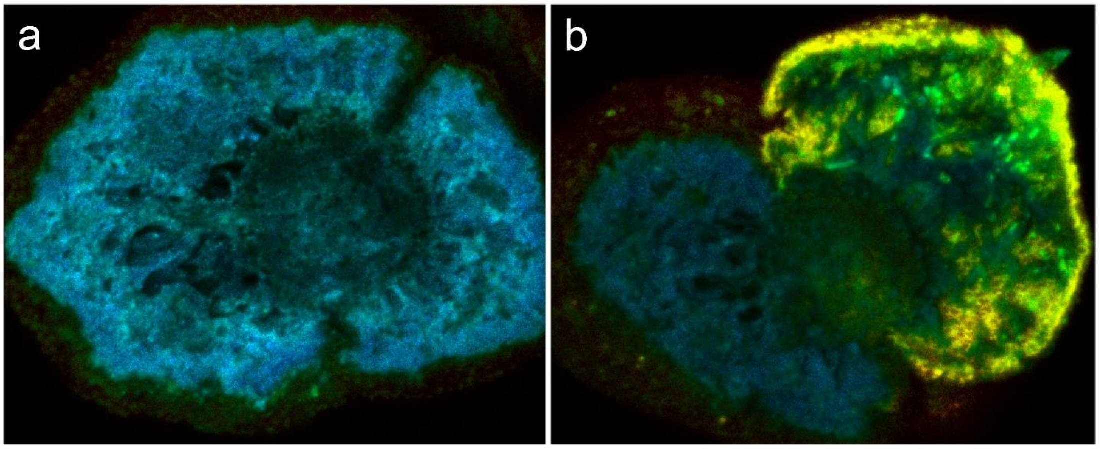

Fig. S11. Evidence for foliar uptake. (a) Microtomed cross-section of a fresh branch showing only autofluorescence in blue color (untreated leaves). (b) Microtomed cross-section of a branch treated with 0.1% lucifer yellow showing diffusion of the dye inside the leaf.

Supporting Information Tables

Table S1. XPS analysis on the *T. aphylla salt* samples PS-3 and PS-4

Sample: PS-3	Position A At%	Position B At%	Position C At%	Sample: PS-4	Position A At%	Position B At%	Position C At%
Li1s	Not detected	Not detected	Not detected	Li1s	Not detected	Not detected	Not detected
Mg2s	1.6	5.9	1.7	Mg2s	3.8	3.9	4.3
Si2p	2.0	5.4	8.2	Si2p	1.4	1.9	2.3
S2p	9.7	2.4	0.7	S2p	1.3	1.4	1.8
Cl2p	1.4	3.6	15.9	Cl2p	14.8	10.0	7.6
C1s	24.1	38.2	28.4	C1s	51.2	55.5	54.2
K2p	0.1	0.3	0.6	K2p	0.1	0.1	0.1
Ca2s	10.7	2.5	1.3	Ca2s	0.4	0.5	0.9
O1s	48.3	40.4	26.7	O1s	18.5	22.0	25.5
Na1s	1.3	1.2	14.3	Na1s	8.5	4.7	3.3

Table S2. Phase identification and quantification using powder X-ray diffraction (PXRD) of five different natural salt samples collected during different times of the year. Sample PS-1 was collected on November 2020, PS-2 was collected on January 2021, PS-3 was collected on February 2021, and PS-4 was collected on May 2021. The samples PS-1 through PS-4 were from the same plant, while PS-5 was collected from a different plant in May 2021

Salt	PS-1	PS-2	PS-3	PS-4	PS-5	Deliquescence point at 20 °C
Halite ($NaCl$)	79.6%	90.0%	61.2%	81.1%	79.4%	~75% [3]
Quartz (SiO_2)	1.7%	0.3%	5.4%	2.0%	2.8%	-
Calcite ($CaCO_3$)	6.6%	1.9%	10.3%	6.3%	7.6%	-
Gypsum ($CaSO_4{\cdot}2H_2O$)	10.3%	3.9%	16.4%	4.7%	4.4%	~99% [3]
Sylvite (KCl)	1.6%	0.3%	2.2%	1.4%	0.0%	~86% [4]
Syngenite ($K_2Ca(SO_4)_2{\cdot}H_2O$)	0.0%	3.6%	2.2%	2.5%	3.8%	>85%
Anhydrite ($CaSO_4$)	0.2%	0.0%	1.3%	0.9%	1.0%	-
Lithium sulfate (Li_2SO_4)	0.0%	0.0%	1.0%	1.1%	1.0%	~60%[a]

[a] Based on the onset of condensation observed in Fig. 5i (see the main text) at 25 °C.

Table S3. Comparison in the phase identification and quantification between the laboratory powder X-ray diffraction (PXRD) and the synchrotron PXRD on three different natural salt samples (PS-3, PS-4 and PS-5)

Salt	PS-3 Lab	PS-3 Synchrotron	PS-4 Lab	PS-4 Synchrotron	PS-5 Lab	PS-5 Synchrotron
Halite (NaCl)	61.2%	58.5%	81.1%	81.0%	79.4%	52.0%
Quartz (SiO_2)	5.4%	6.2%	2.0%	2.6%	2.8%	8.5%
Calcite ($CaCO_3$)	10.3%	12.4%	6.3%	3.2%	7.6%	6.1%
Aragonite ($CaCO_3$)	Not detected	0%	Not detected	0%	Not detected	3.1%
Gypsum ($CaSO_4{\cdot}2H_2O$)	16.4%	13.4%	4.7%	3.5%	4.4%	15.4%
Sylvite (KCl)	2.2%	2.2%	1.4%	2.1%	0.0%	0.0%
Syngenite ($K_2Ca(SO_4)_2{\cdot}H_2O$)	2.2%	0.3%	2.5%	1.5%	3.8%	1.9%
Anhydrite ($CaSO_4$)	1.3%	1.4%	0.9%	1.5%	1.0%	2.4%
Lithium sulfate (Li_2SO_4)	1.0%	0.5%	1.1%	0.5%	1.0%	0.6%
Albite ($AlNaSi_3O_8$)	Not detected	1.6%	Not detected	1.6%	Not detected	4.5%
Dolomite ($CaMg(CO_3)_2$)	Not detected	3.5%	Not detected	2.5%	Not detected	5.5%

Table S4. Mass spectrometry with inductively-coupled plasma and ion chromatography analysis of the composition of samples PS-1, PS-3, PS-4 and PS-5

Ions	PS-1 mg / 104 mg	PS-3 mg / 79.3 mg	PS-4 mg / 103 mg	PS-5 mg / 72 mg
Na^+	30.00	23.23	42.31	24.47
Ca^{2+}	0.88	3.02	1.97	1.67
Mg^{2+}	0.32	0.38	0.49	0.24
K^+	1.73	2.66	3.37	1.59
Cl^-	55.20	35.63	55.22	35.19
SO_4^{2-}	3.32	8.35	5.49	5.81
Li^+	-	9.1×10^{-4}	1.43×10^{-3}	6.2×10^{-4}

Table S5. Deliquescence points of the salts at 20 °C that could be obtained by different binary cation-anion combinations of the ions detected in the *T. aphylla* salt mixture[a]

	Na^+	Ca^{2+}	Mg^{2+}	Li^+	K^+
Cl^-	$NaCl$ 75% RH [3]	[a] $CaCl_2$ 33% RH [3]	[b] $MgCl_2$ 34% RH [3]	$LiCl$ 11% RH [6]	KCl 86% RH [4]
CO_3^{2-}	Na_2CO_3 84% RH [7]	$CaCO_3$ -	$MgCO_3$ -	Li_2CO_3 >85% RH [6]	K_2CO_3 44% RH [4]
SO_4^{2-}	[d] Na_2SO_4 95% RH [3]	[c] $CaSO_4$ 99% RH [3]	$MgSO_4$ 90% RH [5]	~Li_2SO_4 60% RH	K_2SO_4 98% RH [5]

[a]The deliquescence points refer to the following hydrate forms $CaCl_2 \cdot 6H_2O$ (a), $MgCl_2 \cdot 6H_2O$ (b), $CaSO_4 \cdot 2H_2O$ (c) and $Na_2SO_4 \cdot 10H_2O$ (d).

SI References

1. S. Barreca, M. Bruno, L. Oddo, S. Orecchio, Preliminary study on analysis and removal of wax from a Carrara marble statue. *Nat. Prod. Res.* **33**, 947–955 (2019).
2. B. M. Szafranek, E. E. Synak, Cuticular waxes from potato (Solanum tuberosum) leaves. *Phytochem.* **67**, 80–90 (2006).
3. P. Lopez-Arce, E. Doehne, J. Greenshields, D. Benavente, D. Young, Treatment of rising damp and salt decay: the historic masonry buildings of Adelaide, South Australia. *Mater. Struct.* **42**, 827–848 (2009).
4. J. Schönherr, M. Luber, Cuticular penetration of potassium salts: Effects of humidity, anions, and temperature. *Plant Soil* **236**, 117–122 (2001).
5. V. Fernández, T. Sotiropoulos, P. H. Brown, Asociación Internacional de la Industria de los Fertilizantes. Foliar Fertilization: Scientific Principles and Field Practices. (International Fertilizer Industry Association, 2013).
6 C. Peng, L. Chen, M. Tang, A database for deliquescence and efflorescence relative humidities of compounds with atmospheric relevance. *Fundam. Res.* **2**, 578–587 (2022).
7 P. Yang, *et al.,* Hygroscopicity measurement of sodium carbonate, β-alanine and internally mixed β-alanine/Na_2CO_3 particles by ATR-FTIR. *J. Environ. Sci.* **87**, 250–259 (2020).

Captions to the supporting movies

Movie S1 (separate file). Time-lapse video of the *Tamarix aphylla* branches during the diurnal cycle showing the salt crystallization and dissolution cycle. The video was recorded with a plant in its natural habitat in March 2019.

Movie S2 (separate file). Time-lapse video of the collection of humidity by the salt crystals of *Tamarix aphylla*. The video was recorded with a plant in its natural habitat in September 2019.

Movie S3 (separate file). Salt crystallization from ionic excretions on the branches of the *Tamarix aphylla.*
